# Recent advances in the pharmacological effects and mechanisms of borneol and borneol-containing formulations in central nervous system diseases

**DOI:** 10.3389/fphar.2026.1950139

**Published:** 2026-10-29

**Authors:** Xifang Zhang, Yijun Wu, Jin Han, Zicheng Zhao, Yujiao Yao, Yongkang Sun, Xinzhi Wang

**Affiliations:** 1 Encephalopathy Center of the First Affiliated Hospital of Henan University of Chinese Medicine, Zhengzhou, China; 2 The First Clinical Medical College of Henan University of Chinese Medicine, Zhengzhou, China; 3 Collaborative Innovation Center of Prevention and Treatment of Major Diseases by Chinese and Western Medicine, Zhengzhou, China; 4 Institute of Encephalopathy, Henan Academy of Chinese Medical Sciences, Zhengzhou, China

**Keywords:** borneol, borneol-containing formulations, central nervous system diseases, mechanisms of action, pharmacological effects

## Abstract

Borneol is an aromatic medicinal agent widely used in traditional Chinese medicine, both as a standalone component and as an ingredient in multi-herb formulations. Its pharmacological relevance to central nervous system (CNS) disorders has attracted increasing attention, particularly in Alzheimer’s disease, Parkinson’s disease, epilepsy, ischemic stroke, and glioma. Accumulating evidence indicates that borneol may influence multiple pathophysiological processes, including brain-barrier function and drug delivery, neuroinflammation and oxidative stress, neurotransmitter homeostasis and neuronal excitability, cellular injury and death, and the clearance of pathological substances and post-injury repair. Here, we integrate and compare these mechanisms across disease contexts. Notably, although several actions of borneol appear to converge across CNS disorders, others are strongly disease- and context-dependent; for example, its effects on brain-barrier permeability, oxidative stress, and cell death may differ in direction according to disease pathology and therapeutic intent. Pharmacokinetic studies support measurable brain exposure to borneol, yet direct comparative evidence of pharmacological activity, brain disposition, and human pharmacokinetics among borneol preparations of different botanical origins and stereochemical configurations remains scarce. Borneol-containing formulations also exhibit multi-level pharmacological effects; however, because most studies lack borneol-alone, borneol-depleted, or add-back controls, these effects cannot be unequivocally attributed to borneol itself. Overall, the current evidence base remains dominated by cellular and animal studies, with several proposed mechanisms inferred primarily from changes in molecular expression or computational predictions. Robust causal validation, high-quality clinical evidence, and long-term safety data are still limited. Future studies should therefore define the dose–exposure–response relationships and disease-specific therapeutic windows of different borneol preparations, establish the independent contribution of borneol within complex formulations, and determine its genuine therapeutic value and long-term safety through rigorous mechanistic and clinical investigation.

## 1 Introduction

Central nervous system (CNS) diseases are a collective term for disorders caused by pathological changes in the brain and spinal cord, encompassing neurodegenerative diseases, cerebrovascular diseases, brain tumors, and other conditions. According to the Global Burden of Disease 2021 study, neurological disorders have become the leading contributor to the global disease burden, with CNS diseases representing major causes of disability and mortality and imposing an increasingly substantial health burden as populations age (Collaborators, 2024; Zhang C. et al., 2025). Despite this growing burden, the pathogenesis of CNS diseases is highly complex, and therapeutic options for many disorders remain largely limited to symptomatic management or slowing disease progression, with substantial limitations in disease-modifying efficacy and long-term clinical benefit. Moreover, the blood–brain barrier (BBB), while essential for maintaining CNS homeostasis, constitutes a major obstacle to the delivery of exogenous therapeutics into the brain and thus remains a critical challenge in CNS drug development and clinical translation (Rust et al., 2025; Searson and Banks, 2026). Accordingly, therapeutic strategies that simultaneously target multiple pathological processes while improving drug delivery to the brain have become an important direction in CNS pharmacological research.

Borneol, historically known as *Longnao* or *Longnaoxiang* in traditional Chinese medicine, is described in the *Compendium of Materia Medica* (*Bencao Gangmu*) as an agent that “opens the orifices and disperses constrained fire” and has traditionally been used to restore consciousness, clear heat, and relieve pain. The 2025 edition of the *Chinese Pharmacopoeia* includes three forms of borneol—natural borneol, synthetic borneol, and Aipian—whose principal constituents are d-borneol, synthetic borneol, and l-borneol, respectively. Borneol preparations derived from different sources differ in chemical composition and stereochemical configuration (Ma et al., 2023a), and whether these differences translate into equivalent or distinct pharmacological effects requires careful evaluation based on experimental evidence. Among these preparations, synthetic borneol is the most widely used in Chinese patent medicines because of its readily available raw materials and relatively low cost (Jin et al., 2023). Modern pharmacological studies have shown that borneol possesses multiple biological activities, including anti-inflammatory, antioxidant, and anti-apoptotic effects. It can also modulate brain-barrier function and facilitate drug delivery to the brain, while exerting bidirectional regulatory effects on neuronal excitability and its homeostasis (Liu et al., 2021; Hu et al., 2024; Yi et al., 2025). These pharmacological properties have stimulated considerable interest in the potential roles of borneol in neuroprotection and CNS drug delivery.

In recent years, increasing attention has been devoted to the pharmacological effects of borneol in CNS diseases, particularly Alzheimer’s disease (AD), Parkinson’s disease (PD), epilepsy (EP), ischemic stroke (IS), and glioma. These conditions encompass distinct pathological categories, including neurodegenerative diseases, disorders characterized by abnormal neuronal excitability, cerebrovascular diseases, and brain tumors, and currently represent the major disease contexts in which the CNS pharmacology of borneol has been investigated. Available evidence suggests that the effects of borneol involve multiple pathophysiological processes, including brain-barrier function and drug delivery, neuroinflammation and oxidative stress, neurotransmitter homeostasis and neuronal excitability, cellular injury and death, as well as the clearance of pathological substances from the brain and post-injury repair. Although these mechanisms overlap to varying degrees across different CNS diseases, the specific molecular targets, direction of regulation, and resulting biological effects may differ depending on the disease type and pathological context.

Nevertheless, the existing evidence remains fragmented and is predominantly derived from individual disease models or studies focusing on specific molecular pathways. Consequently, the mechanisms shared by borneol across different CNS diseases, as well as their disease-specific differences, have not yet been comprehensively compared from a cross-disease perspective. Furthermore, important distinctions remain insufficiently defined among borneol preparations of different sources and types, borneol administered alone versus in combination with other interventions, and complex borneol-containing formulations. The strength and limitations of the available evidence, as well as its relevance to clinical translation, also require further clarification. Therefore, this narrative review integrates and compares the major pharmacological mechanisms of borneol in CNS diseases, with particular emphasis on both shared mechanisms across diseases and context-dependent differences among distinct pathological conditions. We further compare the pharmacological evidence for different forms of borneol and critically discuss borneol-containing formulations, pharmacokinetic characteristics, the strength and limitations of the available evidence, safety, and challenges in clinical translation. Through this integrative analysis, we aim to provide a clearer framework for mechanistic research on borneol and to inform its rational application in CNS diseases.

## 2 Methods

A structured literature search was conducted in PubMed, Web of Science, Embase, and the China National Knowledge Infrastructure (CNKI) from database inception to 8 September 2026. The initial search was designed to broadly identify studies related to borneol and borneol-containing interventions in the central nervous system (CNS), without prespecifying individual CNS diseases or specific formulations. The search strategy combined subject terms and free-text terms. Borneol-related terms included “borneol,” “Borneolum,” “dexborneol,” and terms describing natural, synthetic, and stereoisomeric forms of borneol. These were combined with broad CNS- and brain-related terms such as “central nervous system,” “CNS,” “brain,” “brain disease,” and “cerebral.” The initial database search yielded 683 records, including 168 from PubMed, 68 from Web of Science, 349 from Embase, and 98 from CNKI. After removal of 185 duplicate records, 498 records remained for title and abstract screening. Based on relevance to borneol or borneol-containing interventions, disease scope, publication type, and relevance to the predefined outcomes of interest, 451 records were excluded, including 195 with limited relevance to borneol interventions, 142 with limited relevance to the predefined outcomes of this review, 94 reviews that did not meet the secondary-evidence inclusion criteria, and 20 studies outside the CNS disease scope of this review. Consequently, 47 reports were retained from the initial search for further assessment. To improve the completeness of evidence retrieval, supplementary targeted searches were subsequently performed. These searches identified 26 additional unique records, including 7 from disease-specific searches, 11 from formulation-specific searches, and 8 from pharmacokinetic, clinical, and safety-related searches. After combining the 47 reports retained from the initial screening with the 26 additional records, 73 reports underwent full-text assessment. No additional reports were excluded according to the eligibility criteria. Finally, 73 studies were included in the qualitative synthesis. The study-selection process is shown in Figure 1.

**FIGURE 1:**
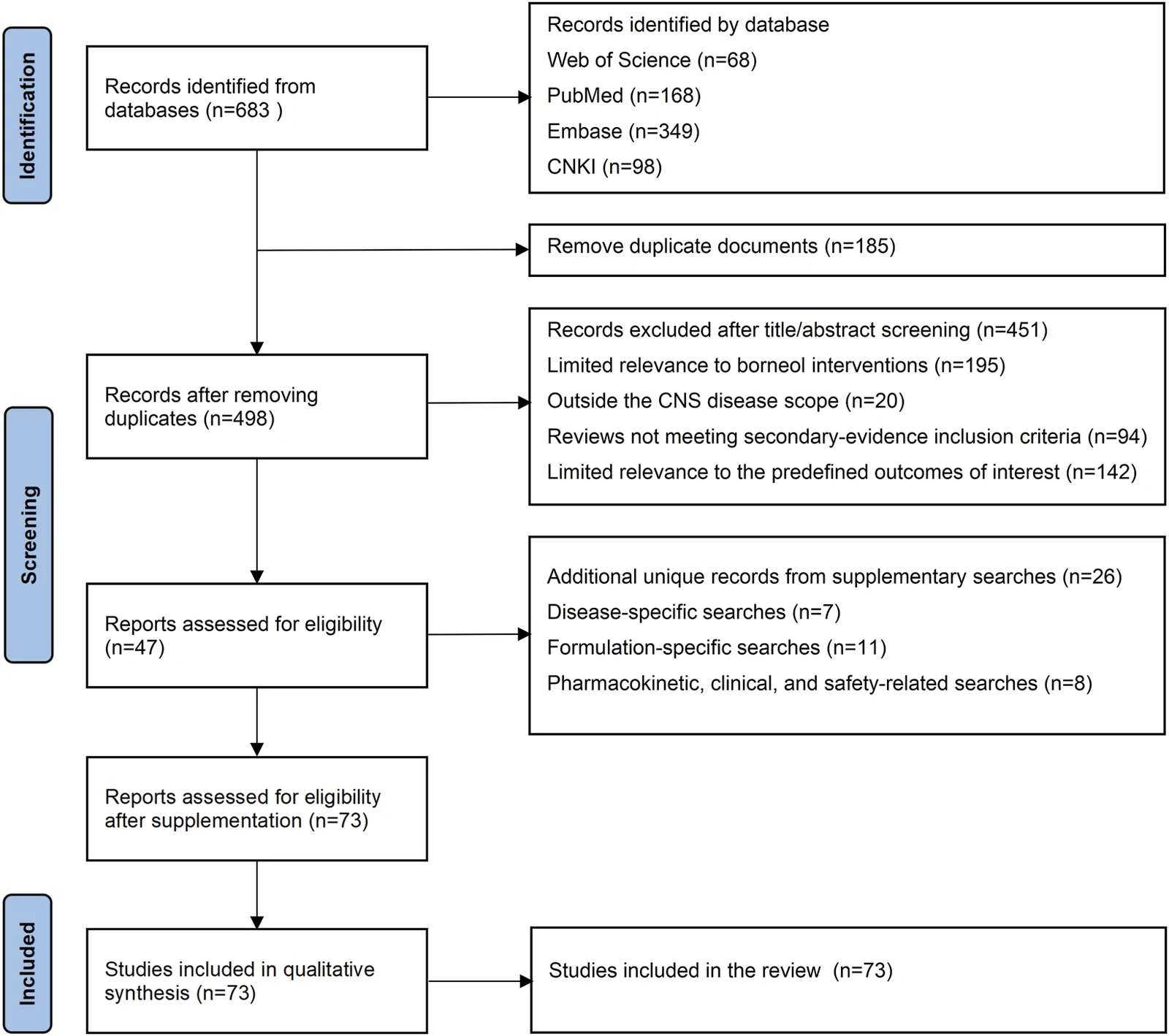
Flowchart of literature identification, screening, and inclusion. Records were identified from PubMed, Web of Science, Embase, and CNKI. After duplicate removal and initial screening, additional relevant studies were identified through supplementary targeted searches. Finally, 73 studies were included in the qualitative synthesis.

## 3 Major mechanisms of borneol in CNS diseases

### 3.1 Brain-barrier function and drug delivery

The blood–brain barrier (BBB) is primarily composed of brain microvascular endothelial cells, pericytes, astrocytic endfeet, and the extracellular matrix, and maintains CNS homeostasis through highly organized tight-junction complexes and selective transport systems (Konig et al., 2025). BBB dysfunction not only contributes to the onset and progression of multiple CNS disorders, including ischemic stroke (IS) and neurodegenerative diseases, but also limits the extent to which therapeutic agents can achieve effective exposure within the brain (Mohi-Ud-Din et al., 2022a; Mohi-Ud-Din et al., 2022b; He et al., 2026). In glioma, the vasculature is further remodeled to form the blood–tumor barrier (BTB). Despite its structural abnormalities and heterogeneous permeability, the BTB remains incompletely disrupted and continues to substantially restrict the delivery of antitumor agents to tumor tissue (Arvanitis et al., 2020; Pinkiewicz et al., 2025).

Available evidence suggests that the effects of borneol on brain barriers depend on both the functional state of the barrier and the therapeutic objective (Tan et al., 2023). Under pathological conditions characterized by BBB disruption, such as cerebral ischemia, borneol appears to predominantly preserve or restore barrier integrity. By contrast, in drug-delivery settings, borneol may enhance drug exposure in the brain or tumor tissue by modulating transmembrane transport processes and barrier permeability. Thus, the influence of borneol on brain-barrier function should not be viewed simply as a nonspecific increase in permeability, but rather as a context-dependent regulatory effect.

#### 3.1.1 Regulation of brain-barrier homeostasis under pathological conditions

Following IS, injury to brain microvascular endothelial cells and disruption of tight junctions lead to pathological increases in BBB permeability, facilitating the entry of blood-derived components into the brain parenchyma and thereby aggravating vasogenic edema and secondary neuronal injury (Ng et al., 2022; Sasannia et al., 2025). Current evidence indicates that, in this pathological context, borneol primarily acts to improve barrier integrity rather than simply increasing BBB permeability.

In a study investigating the effects of *Chuanxiong* and borneol on cerebral ischemia, Song et al. (2023) included a borneol-only treatment group and found that borneol attenuated oxygen–glucose deprivation-induced injury in brain microvascular endothelial cells and increased the expression of the tight-junction proteins ZO-1, occludin, and claudin-5. These changes were associated with HIF-1α/VEGF-related signaling, while combined treatment with *Chuanxiong* and borneol further improved several barrier-related indices. These findings provide direct evidence that borneol itself can contribute to the preservation of tight-junction integrity under ischemic conditions, although the enhanced effects observed with the combination treatment also suggest potential interactions between borneol and other therapeutic components.

Beyond its effects on tight-junction architecture, borneol may also influence post-ischemic BBB function by regulating transcellular transport. Wang et al. (2026) reported that an l-Borneolum preparation modulated the sterol regulatory element-binding protein 1 (SREBP1)/major facilitator superfamily domain-containing protein 2A (MFSD2A)/caveolin-1 (Cav-1) signaling axis. Specifically, l-borneol restored MFSD2A expression and suppressed Cav-1-mediated abnormal vesicular transcytosis, thereby reducing albumin leakage, limiting the pathological increase in BBB permeability after ischemia, and alleviating vasogenic cerebral edema. Zhang et al. (2017) similarly showed that borneol attenuated abnormal BBB opening in a model of focal cerebral ischemia induced by ultrasound and microbubbles, as evidenced by reduced Evans blue extravasation and cerebral edema. Collectively, these findings support a protective role for borneol in maintaining or restoring BBB stability under pathological conditions characterized by barrier disruption.

#### 3.1.2 Brain-barrier modulation and drug delivery to the brain

In contrast to pathological BBB disruption, in drug-delivery settings, borneol may increase the brain exposure of certain therapeutic agents by modulating tight junctions, efflux transporters, and transcellular transport. A preclinical systematic review and meta-analysis by Zheng et al. (2018) showed that co-administration of borneol with other drugs increased the brain exposure of a range of therapeutic agents; however, these findings were derived exclusively from preclinical animal studies. Hui et al. (2024) reported that borneol enhanced the brain distribution of salvianic acid A (SAA), an effect associated with the regulation of P-glycoprotein (P-gp) expression and efflux activity. These findings suggest that attenuation of P-gp-mediated drug efflux may represent one mechanism by which borneol facilitates drug delivery across the BBB. Wang X. et al. (2025) further demonstrated that d-borneol increased edaravone exposure in ischemic brain tissue, accompanied by upregulation of organic anion transporters 1 and 3 (OAT1 and OAT3) and downregulation of P-gp and multidrug resistance-associated protein 1 (MRP1). Transporter-inhibition and target-binding experiments further confirmed the involvement of OAT3 and P-gp in the trans-BBB transport of edaravone, indicating that transporter-mediated transcellular transport may be an important mechanism through which d-borneol enhances drug delivery to the brain.

In glioma, studies have primarily focused on modulation of BTB permeability. In a rat C6 glioma model, Duan et al. (2016) found that natural borneol increased the bioavailability of cisplatin in brain tissue and enhanced BTB permeability through reversible downregulation of ZO-1 and F-actin expression. Xing et al. (2016) established an *in vitro* BTB model by co-culturing C6 glioma cells with human umbilical vein endothelial cells (HUVECs) and observed that natural borneol increased BTB permeability and promoted extracellular signal-regulated kinase (ERK) phosphorylation. These findings suggest that mitogen-activated protein kinase (MAPK)/ERK signaling may contribute to the regulation of BTB permeability by natural borneol.

Taken together, the effects of borneol on brain barriers depend on both barrier status and the therapeutic objective. Under pathological conditions characterized by ischemia-induced BBB disruption, borneol primarily preserves tight-junction integrity, suppresses aberrant transcellular transport, and reduces pathological leakage. By contrast, in drug-delivery settings, borneol can enhance drug exposure in the brain or tumor tissue by modulating transport systems such as P-gp and OAT3 or by increasing BTB permeability. Thus, protection of the pathologically disrupted BBB and enhancement of drug delivery should not be regarded as simply opposing effects; rather, they reflect the context-dependent actions of borneol on distinct barrier states and transport processes. The available evidence on the effects of borneol on brain-barrier function and drug delivery is summarized in Table 1.

**TABLE 1:** Effects of borneol on brain-barrier function and drug delivery.

Refs.	Borneol form (source/purity)	Disease/context	Model	Dose/route/timing	Main finding/mechanism	Study type	Key validation	Notes
Song et al., 2023	Borneol (stereochemistry NS); Beijing Sanhe Pharmaceutical; 99.1%	IS	Primary mouse BMECs with OGD + GCIR mice	0.3 μg/mL; 18-h pretreatment + 6-h OGD (*in vitro*); 160 mg/kg, p.o., once daily ×10 days (7 days pre-GCIR + 3 days post-GCIR) (*in vivo*)	Preserved BBB/TJ integrity; ↑ ZO-1, occludin, claudin-5; ↑ HIF-1α/VEGF-related signaling	*In vivo* + *in vitro*	TEER, NaF permeability, WB of TJ proteins and HIF-1α/VEGF	No pathway-specific validation of HIF-1α/VEGF
Wang et al., 2026	L-Borneolum preparation; Guizhou Miao Medicine Biotechnology; 98%	IS	MCAO/R rats + hCMEC/D3-based BBB co-culture	200 mg/kg, p.o., once daily ×6 days; MCAO 30 min after day-3 dose; l-borneol/L-camphor (6:4) also tested at 200 mg/kg; 50/100/200 μg/mL l-borneol/L-camphor (6:4), 24 h (*in vitro*)	↓ albumin leakage and vesicular transcytosis; ↑ MFSD2A; ↓ Cav-1	*In vivo* + *in vitro* + *in silico*	Albumin leakage, TEM, TEER/HRP, RT-qPCR, WB, network pharmacology	*In vitro* arm used l-borneol/l-camphor; no genetic validation
Zhang et al., 2017	Borneol; form/source/purity NR	IS	Transient MCAO/R mice + UTMD	200 mg/kg, oral gavage, administered 3 days before MCAO; UTMD at 24 h post-MCAO	↓ excessive BBB opening and EB extravasation; ↓ microvascular damage and cerebral edema	*In vivo*	EB extravasation, TEM, brain water content, histology	Molecular mechanism not investigated
Zheng et al., 2018	Pooled borneol preparations; heterogeneous forms/source/purity	CNS drug delivery	Preclinical systematic review/meta-analysis; 58 studies, 1137 animals	Heterogeneous across included studies	↑ CNS drug exposure and BBB permeability across preclinical studies	Preclinical SR/meta-analysis	Quantitative synthesis of CNS drug concentrations and BBB-permeability outcomes	High heterogeneity in form, dose, route, model, and co-treatment
Hui et al., 2024	Borneol (stereochemistry NS); Ji’an Linke Natural Borneol Factory; GC purity >98%	CNS drug delivery	Rats	SBE, SAA-B, or SAA at an equivalent molar dose of 0.136 mmol/kg, i.v.; assessed at 5–60 min	↑ SAA brain exposure; ↓ P-gp expression and efflux activity	*In vivo* + *in silico*	Brain/plasma PK, HPLC, P-gp WB, Rh123 transport assay, virtual screening	Coadministration/prodrug context; no target-specific perturbation
Wang et al., 2025b	D-borneol [(+)-borneol]; Yuan Ye Biotechnology; ≥98%	IS/cerebral drug delivery	MCAO/R rats + OGD/R bEnd.3 cells	0.6 mg/kg alone or 0.6/1.2 mg/kg with edaravone, i.v. (*in vivo*); brain PK assessed over 0.08–24 h; 12.5 μM after 6 h OGD followed by 18 h treatment (*in vitro*)	↑ edaravone exposure in infarcted brain; ↑ OAT1/OAT3; ↓ P-gp/MRP1	*In vivo* + *in vitro* + *in silico*	HPLC-MS/MS, transporter-inhibition experiments, molecular docking, CETSA	Transporter inhibition and CETSA support OAT3/P-gp involvement
Duan et al., 2016	Natural borneol; Lin Ke Natural Borneol Factory, Ji’an; >95% d-borneol	Glioma	C6 glioma-bearing rats	35 or 140 mg/kg, i.g.; cisplatin 10 mg/kg, i.v., 1 h later; assessed at 2 min–8 h	Reversibly ↑ BTB permeability and cisplatin brain exposure; ↓ ZO-1 and F-actin; occludin/claudin-5 unchanged	*In vivo*	Cisplatin brain concentration, qPCR, ELISA, IHC	ZO-1/F-actin association; causal role untested
Xing et al., 2016	Natural borneol; Ji’an Lin Ke Natural Borneol Factory; d-borneol 97.983%	Glioma	C6 cells + HUVEC co-culture BTB model	25/50/100 μg/mL; 0–240 min	Dose- and time-dependent reversible ↑ BTB permeability; ↑ ERK phosphorylation	*In vitro*	HRP permeability assay + WB of ERK/p-ERK, p38/p-p38 and JNK/p-JNK	No ERK-specific causal validation

Study type indicates the original experimental design; Key validation summarizes the principal experimental approaches; Notes highlight major considerations relevant to mechanistic interpretation. ↑/↓ indicate increase/decrease; NR, not reported; NS, not specified.

### 3.2 Neuroinflammation and oxidative stress

Neuroinflammation and oxidative stress are interconnected pathological processes shared by multiple CNS diseases. Sustained release of inflammatory mediators can promote the generation of reactive oxygen species (ROS), whereas excessive oxidative stress can, in turn, activate inflammation-related signaling pathways, thereby establishing a self-reinforcing cycle that exacerbates neuronal injury (Fabisiak and Patel, 2022; Scarian et al., 2024; Dash et al., 2025). Previous studies have shown that various plant extracts and naturally derived compounds can reduce the levels of inflammatory mediators, including tumor necrosis factor-α (TNF-α), interleukin-6 (IL-6), and nitric oxide (NO), while modulating indices associated with oxidative stress (Ahmad et al., 2022; Mir et al., 2023; Mir et al., 2024). In CNS diseases, the effects of borneol on neuroinflammation and oxidative stress primarily involve the regulation of inflammatory mediator release, microglial activation, nuclear factor kappa B (NF-κB)-related signaling, and endogenous antioxidant defense systems.

In AD-related studies, however, the available evidence is derived primarily from essential oil obtained from the borneol chemotype of *Cinnamomum camphora*, a multicomponent volatile extract rather than purified borneol. You et al. (2026) reported that this essential oil reduced the levels of TNF-α, interleukin-1β (IL-1β), and IL-6 in the brains of amyloid precursor protein/presenilin-1 (APP/PS1) mice. It also suppressed extracellular signal-regulated kinase 1/2 (ERK1/2) phosphorylation, inhibitor of nuclear factor kappa B alpha (IκBα) degradation, and NF-κB nuclear translocation, accompanied by attenuation of neuroinflammation and amyloid-β (Aβ) deposition. Tang et al. (2021) further showed that the essential oil increased the activities of superoxide dismutase (SOD) and glutathione peroxidase (GSH-Px), decreased malondialdehyde (MDA) levels, and was accompanied by reduced Aβ deposition and improved cognitive performance.

In PD models, Ding et al. (2024) found that d-borneol reduced the levels of IL-1β, IL-6, and TNF-α in the brains of mice exposed to 1-methyl-4-phenyl-1,2,3,6-tetrahydropyridine (MPTP). It also decreased MDA levels while increasing SOD and GSH-Px activities, accompanied by attenuated dopaminergic neuronal injury in the substantia nigra and striatum. These findings suggest that the neuroprotective effects of d-borneol may be associated with the attenuation of neuroinflammation and oxidative stress.

In epilepsy models, Gao et al. (2023) reported that d-borneol reduced the expression of IL-1β, TNF-α, and cyclooxygenase-2 (COX-2) in pilocarpine-induced epilepsy, suppressed NF-κB signaling activation, and was accompanied by reduced glial activation, neuronal injury, and apoptosis. Li et al. (2024) further showed that d-borneol inhibited pro-inflammatory M1 polarization of microglia, reduced the release of TNF-α and IL-1β, and suppressed activation of the Toll-like receptor 4 (TLR4)/NF-κB signaling pathway. Importantly, administration of a TLR4 agonist partially attenuated these effects, providing additional evidence for the involvement of TLR4/NF-κB signaling in the anti-inflammatory action of d-borneol. In addition, Tambe et al. (2016) found that borneol increased SOD and catalase (CAT) activities and glutathione (GSH) levels, reduced MDA levels, and decreased glial fibrillary acidic protein (GFAP) expression and hippocampal neuronal injury in a pentylenetetrazole-kindled mouse model. These findings suggest that borneol may alleviate epilepsy-associated oxidative stress and glial activation.

In studies of cerebral ischemia, the reported effects of borneol have primarily involved microglial activation and polarization, NF-κB/TLR4-related inflammatory signaling, and endogenous antioxidant defenses. Xie et al. (2022) compared natural borneol with synthetic borneol in cerebral ischemic injury and found that both forms ameliorated ischemia-induced damage to the neurovascular unit while suppressing p65 expression and microglial activation. Dong et al. (2025) reported that Aipian increased total glutathione (T-GSH) and SOD levels in rats subjected to cerebral ischemia–reperfusion, while reducing MDA, TNF-α, and IL-1β levels and downregulating the mRNA expression of Toll-like receptor 4 (TLR4), myeloid differentiation primary response 88 (MyD88), and NF-κB. These findings suggest that Aipian may alleviate oxidative stress and inflammatory responses, potentially through modulation of TLR4/MyD88/NF-κB signaling. Ding et al. (2023) found that borneol modulated M1/M2 microglial polarization after cerebral ischemia–reperfusion, reduced IL-1β levels, and increased interleukin-10 (IL-10) levels, accompanied by suppression of TLR4/MyD88/NF-κB signaling activation. Lu et al. (2023) compared l-borneol, natural borneol, and synthetic borneol and found that all three exhibited anti-inflammatory effects to varying degrees. Among them, l-borneol modulated microglial activation and promoted the expression of M2-associated markers. Xie et al. (2023) further showed, in a subacute rat model of transient middle cerebral artery occlusion (t-MCAO), that l-borneol attenuated inflammatory responses and brain tissue injury, accompanied by alterations in p38 mitogen-activated protein kinase (p38 MAPK)-related signaling and improved neurovascular unit function.

In contrast to the attenuation of inflammation and oxidative injury observed in the non-neoplastic CNS diseases described above, studies in glioma indicate that natural borneol can enhance chemotherapy-induced ROS-mediated damage. Cao et al. (2019), Cao et al. (2020) found that natural borneol potentiated doxorubicin- or cisplatin-induced ROS generation and DNA damage, accompanied by alterations in ataxia telangiectasia mutated/ataxia telangiectasia and Rad3-related protein (ATM/ATR)-p53-p21, mitogen-activated protein kinase (MAPK), and phosphoinositide 3-kinase/protein kinase B (PI3K/Akt)-related signaling, thereby increasing the sensitivity of glioma cells to chemotherapeutic agents. Notably, in the cisplatin study, antioxidant intervention attenuated these signaling alterations and restored cell viability, providing relatively direct evidence that ROS contributes to the chemosensitizing effect of natural borneol. Wang M. L. et al. (2023) found that natural borneol downregulated the expression of the drug-efflux transporters ATP-binding cassette subfamily B member 1 (ABCB1) and ATP-binding cassette subfamily G member 2 (ABCG2) in glioma cells, thereby increasing the cellular uptake and intratumoral accumulation of piperlongumine (PL). This was accompanied by enhanced ROS generation, apoptosis, and G2/M cell-cycle arrest, ultimately potentiating the antiglioma activity of PL.

The effects of borneol on neuroinflammation and oxidative stress vary across different CNS disease contexts. In non-neoplastic disease models, including PD, epilepsy, and cerebral ischemia, borneol predominantly reduces pro-inflammatory mediator levels, suppresses microglial activation, attenuates lipid peroxidation, and enhances endogenous antioxidant defenses, accompanied by changes in NF-κB-, TLR4/MyD88/NF-κB-, and MAPK-related signaling. Similar effects have also been observed in AD-related studies of essential oil from the borneol chemotype of *C. camphora*; however, because this preparation is a multicomponent volatile extract, these effects cannot be directly attributed to borneol itself. In contrast, studies in glioma indicate that natural borneol can enhance ROS-mediated damage, thereby increasing the sensitivity of tumor cells to chemotherapeutic agents. Overall, most mechanistic conclusions in this field are still based primarily on changes in biochemical indices and signaling-protein expression, and the causal relationships underlying these effects require further experimental validation. The disease- and context-dependent effects of borneol on neuroinflammation and oxidative stress are summarized in Table 2.

**TABLE 2:** Modulation of neuroinflammation and oxidative stress by borneol in CNS diseases.

Refs.	Borneol form (source/purity)	Disease	Model	Dose/route/timing	Main finding/mechanism	Study type	Key validation	Notes
You et al., 2026	Essential oil from the borneol chemotype of *Cinnamomum camphora*; Shanghai Tianmei Aromatic Plant Technology; multicomponent essential oil	AD	APP/PS1 mice	3% (v/v) aqueous essential-oil solution; inhalation 1 h/session, twice daily ×21 days	↓ TNF-α, IL-1β, and IL-6; ↓ ERK1/2/NF-κB-related activation	*In vivo* + *in silico*	GC-TOFMS, network pharmacology, molecular docking, WB, ELISA	Multicomponent essential oil; attribution to borneol limited
Tang et al., 2021	Essential oil from the borneol chemotype of *Cinnamomum camphora*; Shanghai Jiao Tong University Aromatherapy Laboratory; borneol 23.833% of 43 identified constituents	AD	APP/PS1 mice + cell-free antioxidant assays	3% (v/v), inhalation 1 h/session, twice daily ×21 days; 2/4/6/8/10 g/L in cell-free assays	↑ SOD and GSH-Px; ↓ MDA	*In vivo* + cell-free assay	DPPH/ABTS assays; brain SOD/GSH-Px/MDA measurements	Multicomponent essential oil; attribution to borneol limited
Ding et al., 2024	D-borneol [(+)-borneol]; Linke Borneol Science and Technology; >98%	PD	MPTP-treated mice	7.5/15/30 mg/kg, i.g., once daily ×30 days; subsequently MPTP 20 mg/kg, i.p., ×5 days	↓ IL-1β, IL-6, TNF-α, and MDA; ↑ SOD and GSH-Px	*In vivo*	ELISA/biochemical assays of inflammatory and oxidative-stress markers	No mediator-specific causal validation
Gao et al., 2023	D-borneol; Simcere Pharmaceutical Group; purity NR	Epilepsy	Pilocarpine-induced SE rats	3/6/12 mg/kg, i.p., once daily ×7 days after SE termination; 12 mg/kg selected for subsequent mechanistic analyses	↓ IL-1β, TNF-α, and COX-2; ↓ GFAP/Iba1 and NF-κB activation	*In vivo*	WB, ELISA, GFAP/Iba1 analyses	No NF-κB-specific causal validation
Li et al., 2024	D-borneol [(+)-borneol]; Simcere Pharmaceuticals; purity NR	Epilepsy	Pilocarpine-induced SE mice + KA-stimulated BV2 microglia	15 mg/kg, i.p., daily ×7 days (*in vivo*); 25 μM after 12 h KA stimulation followed by another 12 h treatment (*in vitro*)	↓ microglial activation and M1 polarization; ↓ TNF-α, IL-1β, and NO; ↓ TLR4/NF-κB activation	*In vivo* + *in vitro*	TLR4 agonist LPS reversal, WB, IF, ELISA, Griess assay	TLR4 agonist reversal supports pathway involvement
Tambe et al., 2016	Borneol (stereochemistry NS); Sigma-Aldrich; 98%	Epilepsy	PTZ-kindled mice	5/10/25 mg/kg, i.p., 30 min before each PTZ injection; 14 PTZ injections over 28 days	↑ SOD, CAT, and GSH; ↓ MDA/LPO and GFAP	*In vivo*	Oxidative-stress assays, GFAP IHC	Pretreatment design; no pathway-specific validation
Xie et al., 2022	Natural borneol and synthetic borneol; Chengdu Chroma-Biotechnology; purity NR	IS	pMCAO rats	Natural borneol: 0.15/0.3/0.6 g/kg; synthetic borneol: 0.05/0.1/0.2 g/kg; i.g.; 3 days pretreatment + 3 days post-surgery treatment	↓ p65 expression and microglial activation	*In vivo*	p65 WB, Iba1 IHC	Different dose ranges across preparations; no pathway-specific perturbation
Dong et al., 2025	Aipian; Guizhou Aiyuan Ecological Pharmaceutical Development; l-borneol >85%	IS	CCA-occlusion CIRI rats	30 mg/kg, i.g., once daily ×7 days before ischemia	↑ T-GSH and SOD; ↓ MDA, TNF-α, and IL-1β; ↓ TLR4/MyD88/NF-κB mRNA	*In vivo*	Oxidative-stress assays, ELISA, qRT-PCR	Aipian-alone group included; pathway evidence mainly at mRNA level
Ding et al., 2023	Borneol (stereochemistry NS); Hubei Junhui; content 86%	IS	CI/R rats + OGD/R primary rat microglia	15 mg/kg, i.g., once daily ×2 days during reperfusion (*in vivo*); 12.5 mg/L, 60 min before OGD/R (*in vitro*); BAP additionally contained AST IV and PNS	↓ M1 and IL-1β; ↑ M2 and IL-10; ↓ TLR4/MyD88/NF-κB; BAP generally produced stronger effects	*In vivo* + *in vitro*	Iba1/iNOS/Arg1 IF, ELISA, qPCR, WB	Borneol-alone and BAP groups included; BAP stronger than borneol alone
Lu et al., 2023	Aipian, natural borneol, and synthetic borneol; Aipian from Luodian County, Guizhou; natural/synthetic borneol from Chengdu Chroma-Biotechnology; purity NR	IS	Acute cerebral I/R rats	Each preparation: 0.05/0.1/0.2 g/kg, i.g.; pretreatment ×3 days + post-model treatment ×1 day	↓ TNF-α at effective doses; Aipian/synthetic borneol ↓ cortical microglial activation; Aipian ↓ iNOS and ↑ Arg1	*In vivo*	Cytokine ELISA, Iba1 IHC, iNOS/Arg1 qPCR	Matched-dose comparison of three preparations; Aipian showed the strongest overall effect
Xie et al., 2023	L-borneol; Luodian County, Guizhou; >85%	IS	t-MCAO rats	0.05/0.1/0.2 g/kg, i.g.; 3 days pretreatment, surgery 0.5 h after the third dose, then daily treatment ×7 days; 0.2 g/kg used for mechanistic analyses	↓ TNF-α, IL-1β, and IL-6; ↑ IL-4; ↑ p-p38/p-ERK-related signaling	*In vivo*	ELISA, qPCR, WB of p38/ERK-related signaling	No pathway-specific causal validation
Cao et al., 2019	Natural borneol; Guangzhou New BenFu Technology; 99%	Glioma	U251/U87 cells + U251 xenograft mice	80 μg/mL NB + 0.4/0.8 μM DOX, 48 h (*in vitro*); 40 mg/kg NB + 2 mg/kg DOX, tail-vein i.v., every other day through day 21 (*in vivo*)	↑ DOX-induced ROS and ROS-mediated DNA damage; altered ERK/Akt-related signaling	*In vivo* + *in vitro*	DCFH-DA ROS assay, GSH rescue, MAPK/PI3K inhibitors, WB	Combination-specific functional validation
Cao et al., 2020	Natural borneol; Guangzhou New BenFu Technology; 99%	Glioma	U251/U87 cells	5–80 μg/mL NB screening; 40 μg/mL NB + 40 μg/mL cisplatin for 48 h in mechanistic experiments; ROS assessed at 6 h	↑ cisplatin-induced ROS and ROS-mediated oxidative damage; altered MAPK/PI3K-Akt signaling	*In vitro*	DCFH-DA, GSH rescue, MAPK/PI3K inhibitors, WB	*In vitro*; combination-specific functional validation
Wang et al., 2023a	Natural borneol; Jiangxi Linke Borneol Technology; 99.6%	Glioma	C6 cells + C6 tumor-bearing mice	12.5–100 μg/mL screen; 100 μg/mL NB + 20 μM PL, 24 h (*in vitro*); 30 mg/kg NB, i.g. + 3.5 mg/kg PL, i.p., once daily ×21 days (*in vivo*)	↑ PL-induced ROS; ROS-dependent chemosensitization	*In vivo* + *in vitro*	ROS imaging/DCFH-DA, NAC rescue	Combination-specific; ROS dependence functionally supported

Study type indicates the original experimental design; Key validation summarizes the principal experimental approaches; Notes highlight major considerations relevant to mechanistic interpretation. For multicomponent essential oils or combination interventions, findings are attributed to the corresponding intervention rather than to purified borneol. ↑/↓ indicate increase/decrease; NR, not reported.

### 3.3 Neurotransmitter homeostasis and neuronal excitability

Neurotransmitter synthesis, release, clearance, and receptor-mediated signaling collectively maintain normal information processing within the CNS (Ryan et al., 2021; Nimgampalle et al., 2023). The nature and pathological significance of neurotransmitter disturbances vary across CNS diseases and may include impaired monoaminergic transmission, imbalance between excitatory and inhibitory neurotransmission, and abnormalities in neurotransmitter-related metabolism (van van Hugte et al., 2023; Zhou et al., 2023; Soares et al., 2024). Available evidence indicates that the effects of borneol on neurotransmitter systems primarily involve dopaminergic, glutamatergic, and GABAergic neurotransmission.

In an AD-related metabolomics study, Tang et al. (2022) used APP/PS1 transgenic mice and found that treatment with essential oil from the borneol chemotype of *C. camphora* altered multiple amino acid-related metabolites in brain tissue, including aspartate, D-serine, and glutamate. These changes involved pathways such as alanine, aspartate, and glutamate metabolism, suggesting that this multicomponent extract may influence metabolic processes associated with excitatory amino acid neurotransmission.

In a PD model, Ding et al. (2024) used *in vivo* brain microdialysis coupled with ultra-high-performance liquid chromatography–tandem mass spectrometry and found that d-borneol increased striatal dopamine (DA) levels and reduced DA turnover in MPTP-treated mice. D-borneol also attenuated the reductions in dopamine transporter (DAT) and vesicular monoamine transporter 2 (VMAT2) expression and enhanced pharmacologically evoked DA release, indicating that d-borneol may ameliorate impaired dopaminergic neurotransmission in the PD model.

Epilepsy-related studies have provided more direct evidence for the regulation of neuronal excitability by borneol. Wang Y. et al. (2023b) compared the anticonvulsant effects of different borneol enantiomers and found that d-borneol exhibited a relatively pronounced antiseizure effect. Electrophysiological recordings in brain slices showed that d-borneol suppressed high-frequency burst firing in subicular neurons and reduced glutamatergic synaptic transmission. *In vivo* calcium fiber photometry further demonstrated that d-borneol attenuated the abnormally enhanced glutamatergic neuronal activity associated with epileptic states. Xu et al. (2025) found that d-borneol positively modulated currents mediated by α1β3γ2L-containing γ-aminobutyric acid type A (GABA_A) receptors, suggesting that enhancement of GABAergic inhibitory transmission may also contribute to its regulation of neuronal excitability. In an earlier study, Tan et al. (2019) reported that natural borneol reduced the protein expression of transient receptor potential vanilloid 1 (TRPV1) and transient receptor potential ankyrin 1 (TRPA1) in the CA1 and CA3 regions of the hippocampus in mice with status epilepticus and increased GABA levels, accompanied by anticonvulsant and neuroprotective effects.

The effects of borneol on neurotransmitter regulation appear to differ across disease contexts. In PD, current evidence has focused primarily on DA release, metabolism, and transporter-related processes. By contrast, epilepsy studies directly implicate both glutamatergic excitatory transmission and GABAergic inhibitory regulation, with supporting evidence from electrophysiological recordings and *in vivo* measurements of neuronal activity. In AD, however, the available evidence remains largely limited to changes in neurotransmitter-related metabolites and metabolic pathways, whereas direct evidence demonstrating effects on synaptic transmission or receptor function is still lacking. The disease-specific effects of borneol on neurotransmitter homeostasis and neuronal excitability are summarized in Table 3.

**TABLE 3:** Effects of borneol on neurotransmitter homeostasis and neuronal excitability.

Refs.	Borneol form (source/purity)	Disease	Model	Dose/route/timing	Main finding/mechanism	Study type	Key validation	Notes
Tang et al., 2022	Essential oil from the borneol chemotype of *Cinnamomum camphora*; Shanghai Jiao Tong University Aromatherapy Laboratory; multicomponent essential oil	AD	APP/PS1 mice	Concentration NR; inhalation, 1 h/session, twice daily ×21 days (*in vivo*)	Altered aspartate, D-serine, and glutamate-related metabolites; altered alanine/aspartate/glutamate metabolism	*In vivo*	GC-TOFMS metabolomics, OPLS-DA, pathway analysis	Multicomponent oil; indirect metabolic evidence
Ding et al., 2024	D-borneol [(+)-borneol]; Linke Borneol Science and Technology; >98%	PD	MPTP-treated mice	7.5/15/30 mg/kg, i.g.; once daily ×30 days; subsequently MPTP 20 mg/kg, i.p., ×5 days (*in vivo*)	↑ striatal DA; ↓ DA turnover; ↑ DAT/VMAT2; ↑ NOM-induced DA release; attenuated RES-induced DA depletion	*In vivo*	Brain microdialysis–UPLC-MS/MS, NOM/RES challenge, WB, RT-qPCR	Pretreatment design; no target-specific validation
Wang et al., 2023b	D-borneol [(+)-borneol], l-borneol [(−)-borneol], and isoborneol; Sigma; purity NR	Epilepsy	MES/PTZ/kindling/KA mouse models + subicular brain slices	10/30/100 mg/kg; i.p., 30 min before seizure testing; 100 mg/kg once daily ×3 days in chronic KA model (*in vivo*); 10/100 μM; 5-min perfusion (*ex vivo*)	D-borneol ↓ high-frequency firing; ↓ sEPSC/mEPSC and glutamatergic transmission; ↓ seizure-associated glutamatergic Ca^2+^ activity	*In vivo* + *ex vivo*	Whole-cell patch clamp, EPSC/IPSC recording, fiber photometry, EEG	Direct functional evidence; target unidentified
Xu et al., 2025	D-borneol [(+)-borneol]; Meryer; purity NR	Epilepsy	MES/6-Hz seizure mouse models + α1β3γ2L-GABA\(_A\)-expressing HEK293 cells	60/80/100 mg/kg (MES) or 156/234/312 mg/kg (6-Hz), i.p.; 30 min before testing (*in vivo*); EC_50_ = 179.79 ± 51.46 μM (*in vitro*)	↑ α1β3γ2L-GABA_*A*_ receptor currents; additive modulation with RTG	*In vivo* + *in vitro*	Whole-cell patch clamp, recombinant GABA_*A*_ receptor assay, isobolographic analysis	Combination study; *in vivo* target dependence untested
Tan et al., 2019	Natural borneol; Jiangxi Linke Borneol Technology; d-borneol ≥98%	Epilepsy	Pilocarpine-induced SE mice	100/400 mg/kg, i.g.; once daily ×5 days before SE induction (*in vivo*)	↓ TRPV1/TRPA1 immunoreactivity; ↑ hippocampal GABA immunoreactivity	*In vivo*	TRPV1/TRPA1/GABA IHC, seizure assessment	Pretreatment design; marker-based evidence only

Study type indicates the original experimental design; Key validation summarizes the principal experimental approaches; Notes highlight major considerations relevant to mechanistic interpretation. ↑/↓ indicate increase/decrease; NR, not reported; NOM, nomifensine; RES, reserpine; EPSC, excitatory postsynaptic current; IPSC, inhibitory postsynaptic current; RTG, retigabine; MES, maximal electroshock seizure; PTZ, pentylenetetrazol; KA, kainic acid.

### 3.4 Regulation of cellular injury and death

Mitochondrial dysfunction and disruption of Ca^2+^ homeostasis can contribute to neural cell injury and apoptosis, whereas processes such as apoptosis and autophagy are also closely involved in glioma cell survival and therapeutic responses (Walters and Usachev, 2023; Kundu et al., 2024; Malone et al., 2025). Available evidence indicates that the effects of borneol on these processes primarily involve the regulation of mitochondrial function, Ca^2+^ homeostasis, and apoptosis, with additional modulation of autophagy reported in glioma.

In cerebral ischemia models, anti-apoptotic effects represent one of the most extensively investigated cytoprotective mechanisms of borneol. Dong et al. (2018) compared the effects of l-borneol, d-borneol, and synthetic borneol in rats subjected to permanent middle cerebral artery occlusion (pMCAO) and found that the protective effects of the three forms were not identical. Specifically, d-borneol and synthetic borneol reduced the B-cell lymphoma 2-associated X protein/B-cell lymphoma 2 (Bax/Bcl-2) ratio in the ischemic penumbra, whereas l-borneol showed relatively greater efficacy in ameliorating overall ischemic brain injury. These findings suggest that different forms of borneol may differ in both their anti-ischemic efficacy and apoptosis-related effects. Zhang et al. (2021) further found that l-borneol reduced the expression of the mitochondrial calcium uniporter (MCU) and isocitrate dehydrogenase 2 (IDH2), downregulated apoptotic protease-activating factor 1 (Apaf-1), Bcl-2-associated agonist of cell death (Bad), and caspase-3, and upregulated Bcl-2 expression. These molecular changes were accompanied by a reduction in cerebral infarct size and improvement in neurological function, suggesting that attenuation of MCU-associated mitochondrial Ca^2+^ dysregulation and the downstream apoptotic cascade may contribute to the neuroprotective effects of l-borneol. Beyond mitochondrial Ca^2+^-related mechanisms, PI3K/Akt- and MAPK-related signaling has also received attention. In a study investigating the combination of *in vitro* cultured Calculus Bovis and borneol, Chen et al. (2023) included an Aipian-only treatment group and found that Aipian attenuated ischemic brain injury and produced an anti-apoptotic molecular profile in rats subjected to middle cerebral artery occlusion followed by reperfusion. This profile was characterized by increased expression of p-PI3K, p-Akt, and Bcl-2, together with reduced Bax and caspase-3 expression and a lower Bax/Bcl-2 ratio. These changes suggest that PI3K/Akt-related signaling may contribute to the effects of Aipian, although pathway-specific causal validation was not performed. Xie et al. (2023) observed in a subacute t-MCAO rat model that l-borneol increased the Bcl-2/Bax ratio and reduced Bax and caspase-3 expression, producing an anti-apoptotic molecular profile during the subacute phase of cerebral ischemia. Overall, studies of cerebral ischemia consistently show changes in apoptosis-related markers that are compatible with an anti-apoptotic effect of borneol. However, the proposed involvement of MCU-related mitochondrial regulation, PI3K/Akt, p38 MAPK, and other signaling pathways is supported mainly by parallel changes in pathway-associated proteins and apoptosis-related markers, with limited pathway-specific causal validation.

Similar neuroprotective effects have also been observed in epilepsy. Li et al. (2024) found that d-borneol attenuated hippocampal neuronal injury and apoptosis in mice with status epilepticus, accompanied by decreased Bax expression and increased Bcl-2 expression, supporting an inhibitory effect of d-borneol on neuronal apoptosis.

In contrast to its suppression of neuronal cell death in cerebral ischemia and epilepsy, studies in glioma indicate that borneol can promote tumor-cell apoptosis or enhance chemotherapy-induced cell death. Liu et al. (2018) found that natural borneol potentiated temozolomide (TMZ)-induced apoptosis in glioma cells and further exacerbated the loss of mitochondrial membrane potential and mitochondrial fragmentation. These effects were accompanied by alterations in Bcl-2 family proteins and caspase-related signaling, suggesting that mitochondrial dysfunction may contribute to the chemosensitizing effect of natural borneol on TMZ. Wang et al. (2020c) further found that borneol itself induced apoptosis in primary human glioma cells in a dose-dependent manner, with downregulation of Bcl-2 and upregulation of Bax and caspase-3. Overexpression of HIF-1α attenuated these effects, while gene-silencing experiments targeting mechanistic target of rapamycin complex 1 (mTORC1) and eukaryotic translation initiation factor 4E (eIF4E) provided further evidence for the involvement of mTORC1/eIF4E/HIF-1α signaling in borneol-induced glioma cell apoptosis.

Autophagy-related studies have focused primarily on the sensitization of glioma to anticancer therapy. Li et al. (2021) found that the combination of borneol and radiotherapy increased autophagosome formation and the expression of Beclin-1 and microtubule-associated protein 1 light chain 3 (LC3) in glioma cells, while reducing the levels of mTORC1, eIF4E, and HIF-1α. Silencing of mTORC1 or eIF4E similarly increased autophagy-related markers and decreased HIF-1α levels, suggesting that autophagy regulation associated with the mTORC1/eIF4E/HIF-1α axis may contribute to the radiosensitizing effect of borneol. Lin et al. (2024) further found that borneol promoted the autophagic degradation of HIF-1α and enhanced the cytotoxicity of TMZ. Pharmacological inhibition of autophagy attenuated borneol-induced HIF-1α degradation, while *in vivo* experiments showed that combined treatment with borneol and TMZ delayed glioma progression. These findings provide further evidence that autophagy-related processes contribute to the chemosensitizing effects of borneol. Taken together, the available evidence supports autophagy as a regulatory process through which borneol may modulate therapeutic sensitivity in glioma, rather than indicating that enhanced autophagy should be equated directly with tumor-cell death.

The effects of borneol on cellular injury and death show clear disease-dependent differences. In non-neoplastic disease models, such as cerebral ischemia and epilepsy, borneol predominantly attenuates neuronal apoptosis, with mitochondrial Ca^2+^ homeostasis emerging as a potentially important regulatory process in cerebral ischemia. By contrast, in glioma, borneol promotes tumor-cell apoptosis and modulates autophagy-related processes, thereby enhancing sensitivity to radiotherapy and chemotherapy. The disease-dependent effects of borneol on cellular injury and death are summarized in Table 4.

**TABLE 4:** Effects of borneol on the regulation of cellular injury and death.

Refs.	Borneol form (source/purity)	Disease	Model	Dose/route/timing	Main finding/mechanism	Study type	Key validation	Notes
Dong et al., 2018	L-borneol, d-borneol, and synthetic borneol; Luodian (l-borneol) and Chengdu Chroma-Biotechnology (d-borneol and synthetic borneol); purity NR	IS	pMCAO rats	0.2 g/kg (l-/d-borneol) or 0.6 g/kg (synthetic borneol), p.o., once daily ×3 days; pMCAO 30 min after final dose (*in vivo*)	D-borneol and synthetic borneol ↓ Bax/Bcl-2 mRNA ratio; l-borneol showed no significant ratio reduction	*In vivo*	RT-PCR, Bax/Bcl-2 IHC	Form-specific effects; pretreatment design
Zhang et al., 2021	L-borneol; Luodian County, Guizhou; 95%	IS	pMCAO rats	0.05/0.1/0.2 g/kg, p.o.; 2-day pretreatment + 1-day post-pMCAO treatment (*in vivo*)	↓ MCU and IDH2; ↓ Apaf-1, Bad, and caspase-3; ↑ Bcl-2	*In vivo*	RT-qPCR, WB of MCU/IDH2 and apoptosis-related proteins	No MCU-specific causal validation
Chen et al., 2023	Aipian; Guizhou Luodian Ainaxiang Pharmaceutical; purity NR	IS	MCAO/R rats	0.05/0.1/0.2 g/kg, p.o.; 3-day pretreatment + 3-day post-reperfusion treatment; 0.2 g/kg used for mechanistic analyses (*in vivo*)	↑ p-PI3K/p-Akt and Bcl-2; ↓ Bax, caspase-3, and Bax/Bcl-2 ratio	*In vivo*	qRT-PCR, WB, IHC of PI3K/Akt and apoptosis-related proteins	Aipian-only arm; no pathway-specific validation
Xie et al., 2023	L-borneol; Luodian County, Guizhou; >85%	IS	t-MCAO rats	0.05/0.1/0.2 g/kg, i.g.; 3 days pretreatment, surgery 0.5 h after the third dose, then daily treatment ×7 days; 0.2 g/kg used for mechanistic analyses (*in vivo*)	↑ Bcl-2/Bax ratio and p-p38/p-ERK; ↓ Bax and caspase-3	*In vivo*	WB, qPCR of p38/ERK and apoptosis-related markers	No direct apoptosis assay or pathway perturbation
Li et al., 2024	D-borneol [(+)-borneol]; Simcere Pharmaceuticals; purity NR	Epilepsy	Pilocarpine-induced SE mice + KA-stimulated BV2 microglia	15 mg/kg, i.p., daily ×7 days (*in vivo*); 25 μM after 12 h KA stimulation followed by another 12 h treatment (*in vitro*)	↓ Bax; ↑ Bcl-2; attenuated hippocampal neuronal injury	*In vivo* + *in vitro*	Bax/Bcl-2 IF, NeuN WB, TLR4 agonist LPS reversal	Marker-based apoptosis evidence; LPS reversal included
Liu et al., 2018	Natural borneol; BenFu Technology; purity NR	Glioma	U251/U87/BV-2 cells + nude mice	40/80 μg/mL NB + 20/40 μM TMZ, 48 h (*in vitro*); 20 mg/kg/day NB ± 10 mg/kg/day TMZ, daily ×2 weeks; route NR (*in vivo*)	↑ TMZ-induced apoptosis; ↓ MMP; ↑ mitochondrial fragmentation; ↑ Bax/Bad and caspase activation; ↓ Bcl-2/Bcl-XL	*In vivo* + *in vitro*	Flow cytometry, Annexin V–PI/TUNEL, JC-1, Mito-Tracker, WB	Combination-specific; no target-specific perturbation
Wang et al., 2020c	Borneol; form/source/purity NR	Glioma	Primary human glioma cells + C6 glioma rats	10/20/40/80 μg/mL, 24 h (*in vitro*); 4/8/16 mg/kg, once daily ×10 days; route NR (*in vivo*)	Dose-dependent ↑ apoptosis; ↓ Bcl-2, HIF-1α, mTORC1, and eIF4E; ↑ Bax and caspase-3	*In vivo* + *in vitro*	Flow cytometry, HIF-1α overexpression rescue, mTORC1/eIF4E siRNA, WB, qRT-PCR	Genetic validation supports pathway involvement
Li et al., 2021	Borneol; form/source/purity NR	Glioma	Primary human glioma cells + C6 glioma rats	10/20/40/80 μg/mL after 5 Gy irradiation, 48 h; 20 μg/mL used for mechanistic analyses (*in vitro*); 16 mg/kg; 7 days pre-RT + 3 days post-RT; route NR (*in vivo*)	↑ autophagosomes, Beclin-1, and LC3; ↓ mTORC1/eIF4E/HIF-1α; enhanced radiosensitivity	*In vivo* + *in vitro*	TEM, WB/IF, mTORC1/eIF4E silencing, HIF-1α manipulation	Radiotherapy combination; genetic validation included
Lin et al., 2024	Borneol (stereochemistry NS); Shanghai Yuanye Biotechnology; purity NR	Glioma	C6/U251 cells + C6 orthotopic rats	40 μg/mL borneol + TMZ 200 μM (U251) or 250 μM (C6), 48 h (*in vitro*); 16 mg/kg borneol ± 50 mg/kg TMZ, i.g., daily ×9 days (*in vivo*)	↑ autophagic HIF-1α degradation; ↑ Beclin-1/LC3-II/I; enhanced TMZ sensitivity	*In vivo* + *in vitro*	CQ autophagy-inhibitor reversal, TEM, WB, IHC	TMZ combination; autophagy dependence supported

Study type indicates the original experimental design; Key validation summarizes the principal experimental approaches; Notes highlight major considerations relevant to mechanistic interpretation. For combination interventions, findings are interpreted in the context of the corresponding combined treatment unless borneol-specific effects were independently evaluated. ↑/↓ indicate increase/decrease; NR, not reported; NS, not specified; MMP, mitochondrial membrane potential; CQ, chloroquine.

### 3.5 Clearance of pathological substances and post-injury repair

Drainage through meningeal lymphatic vessels (mLVs) contributes to the clearance of waste products and macromolecules from the brain, whereas angiogenesis and neurogenesis are important components of tissue repair following cerebral ischemia (Fang et al., 2023; Paro et al., 2023; Wang M. et al., 2024). Current studies of borneol in this context have focused primarily on meningeal lymphatic drainage in AD and neurovascular remodeling during recovery from cerebral ischemia.

In an AD model, Wu Y. et al. (2023b) investigated orally administered borneol micelles (BO-Ms) and found that treatment increased the diameter of mLVs and enhanced lymphatic drainage. These effects were accompanied by reduced meningeal NO levels and increased expression of forkhead box C2 (FOXC2), vascular endothelial growth factor C (VEGFC), and lymphatic vessel endothelial hyaluronan receptor 1 (LYVE-1), together with enhanced transport of Aβ through mLVs to the deep cervical lymph nodes. These findings suggest that BO-Ms may facilitate Aβ clearance by improving meningeal lymphatic drainage. Ye et al. (2024) further investigated the regulation of lymphatic valves in mLVs using borneol nanomicelles (BO-Ms). BO-Ms increased Akt and FOXC2 expression while decreasing forkhead box O1 (FOXO1) and PR domain zinc finger protein 1 (PRDM1) expression, accompanied by enhanced lymphatic valve formation and improved mLV drainage, thereby facilitating Aβ clearance. Notably, prophylactic administration produced greater overall effects than therapeutic administration initiated after model establishment.

During recovery from cerebral ischemia, Ma et al. (2021) found that l-borneol promoted angiogenesis in ischemic brain tissue, accompanied by changes in angiopoietin-1 (Ang1), vascular endothelial growth factor (VEGF), and brain-derived neurotrophic factor (BDNF). Further studies showed that both l-Borneolum and Borneolum preparations promoted post-ischemic neurogenesis, with the l-Borneolum preparation exerting a more pronounced effect on astrocyte-to-neuron transdifferentiation. These effects were accompanied by changes in Wnt- and Notch-related signaling (Ma et al., 2023b).

The effects of borneol on pathological-substance clearance and post-injury repair appear to be disease-specific. In AD, the available evidence primarily involves mLV-mediated Aβ clearance, whereas in cerebral ischemia, the reported effects are mainly associated with angiogenesis and neurogenesis during the recovery phase. The effects of borneol on pathological-substance clearance and post-injury repair are summarized in Table 5. A broader cross-disease comparison of major mechanistic effects of borneol and borneol-related interventions in CNS diseases is provided in Table 6. The major pharmacological mechanisms of borneol across CNS diseases are summarized in Figure 2.

**TABLE 5:** Effects of borneol on pathological-substance clearance and post-injury repair.

Ref.	Borneol form (source/purity)	Disease	Model	Dose/route/timing	Main finding/mechanism	Study type	Key validation	Notes
Wu et al., 2023b	Borneol micelles (BO-Ms); YuanCheng Saichuang Technology; purity NR	AD	Aβ_42_-injected mice + MLECs	10/20/50 mg/kg, p.o., single-dose Aβ-clearance assays; 20 mg/kg, p.o., once daily ×14 days after Aβ_42_ injection (*in vivo*); 1–50 μg/mL, 24 h or 20/50 μg/mL, 6 h (*in vitro*)	↑ mLV diameter and drainage; ↑ Aβ efflux to dCLNs; ↓ meningeal NO; ↑ LYVE-1/FOXC2/VEGFC	*In vivo* + *in vitro*	dCLN afferent lymphatic ligation, fluorescence imaging, Aβ_42_ ELISA, IF, WB, RT-qPCR, MLEC assays	Micellar formulation; mLV dependence supported
Ye et al., 2024	Borneol nanomicelles (BO-Ms); YuanCheng Saichuang Technology; >85%	AD	Aβ_42_-injected mice	20 mg/kg, i.g., once daily ×10/20/30 days before Aβ_42_ injection or ×30 days after Aβ_42_ injection (*in vivo*)	↑ lymphatic valve formation and mLV drainage; ↑ Akt/FOXC2; ↓ FOXO1/PRDM1; ↑ Aβ clearance	*In vivo*	LYVE-1/FOXC2 IF, confocal imaging, RT-qPCR, WB, OVA-FITC/Aβ_42_ clearance imaging	Prevention > treatment; no pathway-specific perturbation
Ma et al., 2021	D-borneol; Luodian County, Guizhou; 88.6%	IS	pMCAO rats	0.05/0.1/0.2 g/kg, i.g., for 3 days after pMCAO (*in vivo*)	↑ VEGF/Ang1/CD34 and BDNF; ↓ Tie2/TGF-β1/MMP9; promoted angiogenesis/neurogenesis	*In vivo* + *in silico*	ELISA, qRT-PCR, CD34/VEGFR IHC, histology, molecular docking	Marker-based repair evidence; docking not causal
Ma et al., 2023b	L-Borneolum (mainly l-borneol) and Borneolum (mainly d-borneol); Chengdu Croma Biotechnology and Luodian County, Guizhou, respectively; l-borneol 91.30% and d-borneol 97.88%	IS	t-MCAO rats	50/100/200 mg/kg, i.g., 2 h after surgery, once daily ×14 days; 200 mg/kg ×28 days for AAV fate-tracing analyses (*in vivo*)	↑ astrocyte-to-neuron transdifferentiation and neurogenesis; L-Borneolum showed greater neuronal transdifferentiation; altered Wnt/Notch-related proteins	*In vivo*	AAV-GFAP fate tracing, BrdU labeling, IF co-localization, WB of Wnt/Notch-related proteins, LSCI	No gene perturbation or electrophysiological validation

Study type indicates the original experimental design; Key validation summarizes the principal experimental approaches; Notes highlight major considerations relevant to mechanistic interpretation. For micellar formulations, findings are attributed to the formulation rather than to unformulated borneol. ↑/↓ indicate increase/decrease; NR, not reported; dCLNs, deep cervical lymph nodes; MLECs, mouse lymphatic endothelial cells; LSCI, laser speckle contrast imaging.

**TABLE 6:** Cross-disease comparison of major mechanistic effects of borneol and borneol-related interventions in CNS diseases.

Mechanistic domain	IS	AD	PD	Epilepsy	Glioma
Brain-barrier function and drug delivery	Borneol, l-Borneolum, and d-borneol: preserved BBB integrity; ↓ leakage/transcytosis; ↑ TJ proteins; ↑ cerebral drug exposure through transporter modulation (Song et al., 2023; Wang et al., 2026; Zhang et al., 2017; Wang et al., 2025b)	NR	NR	NR	Natural borneol: reversible ↑ BTB permeability and drug exposure; ↓ ZO-1/F-actin; ↑ ERK phosphorylation (Duan et al., 2016; Xing et al., 2016)
Neuroinflammation and oxidative stress	Natural borneol, synthetic borneol, Aipian, l-borneol, and borneol: ↓ pro-inflammatory mediators and microglial activation; M1→M2 shift; ↑ antioxidant defenses; ↓ MDA (Xie et al., 2022; Dong et al., 2025; Ding et al., 2023; Lu et al., 2023; Xie et al., 2023)	Multicomponent essential oil from the borneol chemotype of C. camphora: ↓ TNF-α/IL-1β/IL-6; ↑ SOD/GSH-Px; ↓ MDA (You et al., 2026; Tang et al., 2021)	D-borneol: ↓ IL-1β/IL-6/TNF-α and MDA; ↑ SOD/GSH-Px (Ding et al., 2024)	D-borneol and borneol: ↓ IL-1β/TNF-α/COX-2/NF-κB and microglial activation; ↑ SOD/CAT/GSH; ↓ MDA (Gao et al., 2023; Li et al., 2024; Tambe et al., 2016)	Natural borneol: ↑ ROS and oxidative damage, contributing to chemosensitization (Cao et al., 2019; Cao et al., 2020; Wang et al., 2023a)
Neurotransmitter homeostasis and neuronal excitability	NR	Multicomponent essential oil from the borneol chemotype of C. camphora: altered aspartate/D-serine/glutamate-related metabolism; no direct synaptic evidence (Tang et al., 2022)	D-borneol: ↑ striatal DA; ↓ DA turnover; ↑ DAT/VMAT2; ↑ evoked DA release (Ding et al., 2024)	D-borneol and natural borneol: ↓ glutamatergic transmission/activity; ↑ GABA_*A*_ receptor currents; ↑ hippocampal GABA immunoreactivity (Wang et al., 2023b; Xu et al., 2025; Tan et al., 2019)	NR
Regulation of cellular injury and death	L-borneol, d-borneol, synthetic borneol, and Aipian: predominantly anti-apoptotic; ↓ Bax/Bcl-2 or ↑ Bcl-2/Bax; ↓ caspase-3; MCU/IDH2-associated mitochondrial regulation (Dong et al., 2018; Zhang et al., 2021; Chen et al., 2023; Xie et al., 2023)	NR	NR	D-borneol: ↓ Bax; ↑ Bcl-2; attenuated neuronal injury (Li et al., 2024)	Natural borneol and borneol: ↑ tumor-cell apoptosis and mitochondrial dysfunction; ↑ autophagy-related activity associated with radio-/chemosensitization (Liu et al., 2018; Wang et al., 2020c; Li et al., 2021; Lin et al., 2024)
Clearance of Pathological Substances and Post-Injury Repair	L-borneol and Borneolum/l-Borneolum preparations: ↑ angiogenesis and neurogenesis; ↑ astrocyte-to-neuron transdifferentiation during recovery (Ma et al., 2021; Ma et al., 2023b)	BO-Ms: ↑ mLV drainage and lymphatic valve formation; ↑ Aβ clearance to dCLNs (Wu et al., 2023b; Ye et al., 2024)	NR	NR	NR

↑/↓ indicate increase/decrease; NR, not reported in the included studies. Findings involving multicomponent essential oils or micellar formulations are attributed to the corresponding intervention rather than to purified borneol alone.

**FIGURE 2:**
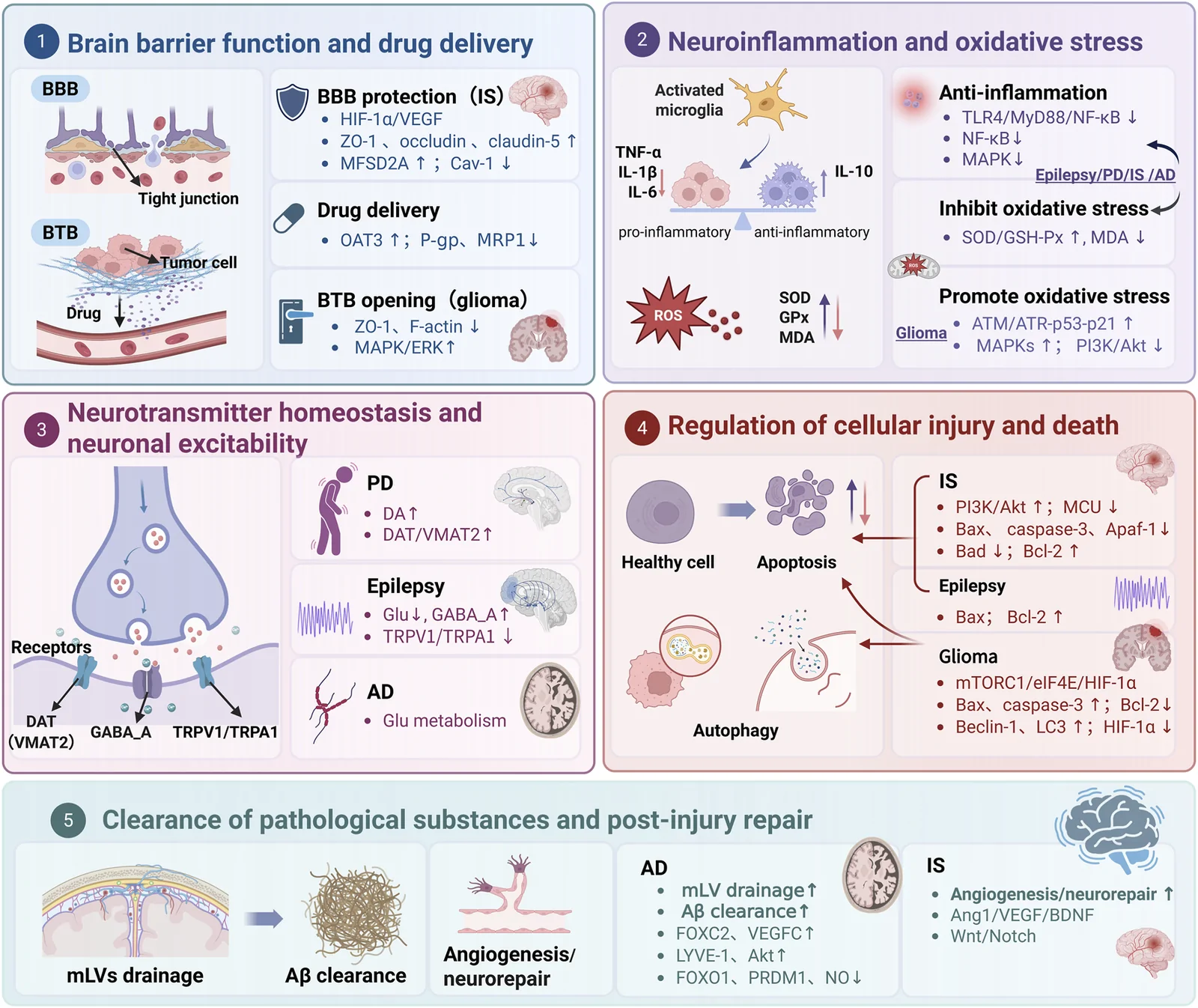
Major mechanisms of borneol in CNS diseases: A cross-disease comparison. ↑/↓ indicate increased/decreased levels. Created in BioRender. zhang, X. (2026) https://BioRender.com/wjdnvml

## 4 Pharmacological effects of borneol-containing formulations in CNS diseases

Borneol is widely incorporated into multi-component formulations, including Compound Danshen Tablets/Dripping Pills, Angong Niuhuang Pills, Suhexiang Pills, Xingnaojing Injection, and Compound Shexiang Injection. Available studies indicate that borneol-containing formulations may exert pharmacological effects in CNS diseases through multiple processes, including modulation of neurotransmission, inflammation and oxidative stress, BBB function, and cellular injury and death. However, all of these preparations are complex multi-component systems. In the absence of borneol-only groups, borneol-depleted controls, or component-deconstruction comparisons, the observed effects primarily reflect the pharmacological actions of the formulations as a whole and cannot be directly attributed to borneol itself. Considerable heterogeneity also exists across studies with respect to the preparations investigated, disease models, routes of administration, and the extent of mechanistic validation.

### 4.1 Studies of Compound Danshen Tablets/Dripping Pills in AD and epilepsy

Compound Danshen Tablets/Dripping Pills consist of *Salvia miltiorrhiza*, *Panax notoginseng*, and borneol, and the available studies have focused primarily on AD and epilepsy. Early animal studies showed that Compound Danshen Tablets increased cortical glycine (Gly) and GABA levels while reducing glutamate (Glu) levels in rat models of AD. These changes were accompanied by reduced Aβ accumulation and improvements in learning and memory, suggesting that the formulation may modulate amino acid neurotransmitter homeostasis and AD-related pathological processes (Qin et al., 2004; Qin et al., 2005). In epilepsy models, Compound Danshen Dripping Pills were reported to suppress the Janus kinase (JAK)/signal transducer and activator of transcription 3 (STAT3) pathway, reduce the p-JAK/JAK and p-STAT3/STAT3 ratios, and decrease TNF-α and IL-6 levels, accompanied by attenuated seizures and hippocampal neuronal injury (Zhao and Liu, 2020). Huang et al. (2022) further used network pharmacology and molecular docking to predict that constituents such as quercetin, luteolin, and tanshinone IIA may act on targets including AKT serine/threonine kinase 1 (AKT1), tumor protein p53 (TP53), IL-6, and TNF and may involve NF-κB-related pathways. However, these findings are based primarily on computational predictions and have not yet been validated by functional experiments targeting the proposed molecules or pathways. Overall, the direct evidence for Compound Danshen Tablets in AD is derived mainly from relatively early animal studies, while more recent experimental and clinical evidence for this indication remains limited.

### 4.2 Studies of Angong Niuhuang Pills in IS

Studies investigating Angong Niuhuang Pills have focused primarily on IS. Wang L. H. et al. (2024b) combined network pharmacology with animal experiments and found that Angong Niuhuang Pills attenuated brain tissue injury and improved neurological function in rats with CIRI. These effects were accompanied by increased expression of mitogen-activated protein kinase 3 (MAPK3) and sirtuin 1 (SIRT1), as well as decreased expression of cell division cycle 42 (CDC42) and poly(ADP-ribose) polymerase 1 (PARP1), suggesting that the therapeutic effects of Angong Niuhuang Pills may involve regulation of inflammation, oxidative stress, and apoptosis. Tsoi et al. (2019) further demonstrated that Angong Niuhuang Pills reduced the levels of p47phox, inducible nitric oxide synthase (iNOS), and 3-nitrotyrosine (3-NT), inhibited abnormal activation of matrix metalloproteinase-2/matrix metalloproteinase-9 (MMP-2/MMP-9), preserved ZO-1 and claudin-5 expression, and improved Bcl-2/Bax-related apoptotic changes. These effects collectively contributed to the attenuation of BBB disruption and neuronal injury following cerebral ischemia–reperfusion. This study also evaluated safety profiles and found that short-term administration of 1–4 times the equivalent dose for 7 days did not result in obvious abnormalities in hepatic or renal function or tissue morphology. However, repeated or higher-dose administration increased arsenic and mercury accumulation *in vivo*, suggesting that the long-term safety of high-dose or prolonged use requires further attention. Zhang X. et al. (2025b) screened a bioequivalent combination component system (BECCs V) derived from Angong Niuhuang Pills and observed comparable protective effects to the original formulation in zebrafish and MCAO mouse models. These findings suggested that signaling pathways including phosphoinositide 3-kinase/protein kinase B/mechanistic target of rapamycin (PI3K/Akt/mTOR), PI3K/Akt/hypoxia-inducible factor-1α (HIF-1α), and PI3K/Akt/forkhead box O3a (FoxO3a) may participate in the regulation of neuronal autophagy, microglial inflammation, and brain microvascular endothelial function. Wang L. H. et al. (2025a) further found that pretreatment with Angong Niuhuang Pills increased SIRT1 expression, reduced histone H4 lysine 16 acetylation (H4K16ac), and inhibited ATG5 and ATG7 expression, accompanied by attenuation of excessive autophagy and neuronal apoptosis. The SIRT1 inhibitor EX527 partially abolished these effects, providing more direct evidence supporting the involvement of the SIRT1–H4K16ac axis in the pharmacological actions of Angong Niuhuang Pills.

### 4.3 Studies of Suhexiang Pills in AD and IS

CNS-related studies of Suhexiang Pills have mainly focused on AD and IS. In an AD model, Jeon et al. (2011) administered a multi-component volatile oil preparation derived from Suhexiang Pills through preventive inhalation and observed increased expression of nuclear factor erythroid 2-related factor 2 (Nrf2) and heme oxygenase-1 (HO-1), together with reduced activation of c-Jun N-terminal kinase (JNK), p38, and Tau phosphorylation. These molecular changes were accompanied by improvements in oxidative stress, neuronal apoptosis, and memory impairment. In an IS model, Zhang et al. (2024) found that Suhexiang Pills increased cyclic adenosine monophosphate (cAMP) and phosphorylated protein kinase A (p-PKA) levels while reducing TNF-α, IL-1β, Bax, and cleaved caspase-3 expression. These changes were accompanied by reduced infarct size and improved neurological outcomes. Furthermore, inhibition of cAMP signaling using the cAMP inhibitor SQ22536 attenuated the anti-inflammatory and anti-apoptotic effects of Suhexiang Pills, providing evidence for the involvement of cAMP/PKA signaling in its protective effects. Li Z. et al. (2025b) further found that Suhexiang Pills increased miR-24-3p expression in neurons subjected to oxygen–glucose deprivation/reoxygenation (OGD/R) injury, inhibited Kelch-like ECH-associated protein 1 (Keap1), and activated Nrf2/HO-1-related signaling, thereby reducing neuronal injury, inflammatory responses, and apoptosis. Suppression of miR-24-3p weakened these protective effects against neuronal injury, inflammation, and apoptosis. Compared with studies based solely on molecular expression changes, this study provided stronger mechanistic support for the involvement of the miR-24-3p/Keap1/Nrf2 axis through gain- and loss-of-function approaches.

### 4.4 Studies of Xingnaojing Injection in AD and IS

Xingnaojing Injection is composed of musk, *Curcuma aromatica* rhizome, borneol, and *Gardenia jasminoides* fruit, and related studies have mainly focused on AD and IS. In AD-related research, Wang M. X. et al. (2020a) integrated microarray analysis with network pharmacology and identified 5-hydroxytryptamine receptor 2A (HTR2A) and adrenoceptor alpha 2A (ADRA2A) as potential target genes associated with the intervention of Xingnaojing Injection in AD, with involvement of the neuroactive ligand–receptor interaction pathway. However, the qRT-PCR validation in this study was primarily used to confirm the expression of AD-associated candidate differentially expressed genes rather than to directly verify the pharmacological effects of Xingnaojing Injection on these targets. Therefore, these findings should still be considered as predictive evidence rather than direct mechanistic validation. Liu et al. (2020) found in an Aβ_1-42_-induced AD mouse model that Xingnaojing Injection enhanced AKT/mTOR-related signaling and NMDA receptor-mediated synaptic transmission, improved dendritic spine density and long-term potentiation, and ameliorated cognitive deficits. The protective effects were attenuated by rapamycin treatment, providing relatively direct evidence supporting the involvement of AKT/mTOR signaling in the regulation of synaptic plasticity by Xingnaojing Injection. In IS models, studies of Xingnaojing Injection have mainly focused on BBB protection, inflammatory regulation, and attenuation of neuronal injury. Qu et al. (2019) found that Xingnaojing Injection inhibited NLRP3 inflammasome activation and preserved ZO-1 and occludin expression, accompanied by improved BBB permeability and reduced brain tissue injury. Zhang et al. (2020) further used the SIRT1 inhibitor EX527 and provided additional evidence supporting the involvement of SIRT1 in the anti-inflammatory effects of Xingnaojing Injection. Liu et al. (2022) combined network pharmacology, a tMCAO animal model, and gut microbiota analysis and found that Xingnaojing Injection reduced TLR4, MyD88, and NF-κB expression in ischemic brain tissue and improved BBB permeability, accompanied by alterations in intestinal barrier-related and microbiota-associated parameters. Cao et al. (2024) further observed in a pretreatment study that Xingnaojing Injection reduced HMGB1/NLRP3-related pyroptosis markers. Dong et al. (2024) reported that Xingnaojing Injection alleviated neuronal Ca^2+^ overload and endoplasmic reticulum stress, accompanied by improvements in apoptosis-related indicators.

### 4.5 Studies of Compound Shexiang Injection in IS

Studies of Compound Shexiang Injection have focused primarily on IS, although the available experimental evidence supporting its mechanisms of action remains relatively limited. Li et al. (2023) used network pharmacology and molecular docking to predict that PI3K/Akt, Rap1, and MAPK signaling may represent relevant pathways, with SRC proto-oncogene, non-receptor tyrosine kinase (SRC), phosphatidylinositol-4,5-bisphosphate 3-kinase catalytic subunit alpha (PIK3CA), and MAPK3 identified as potential key targets. Subsequent experiments in HT-22 cells subjected to OGD/R injury showed trends toward increased PIK3CA and MAPK3 mRNA expression and decreased SRC mRNA expression, together with a trend toward improved cell viability. These findings provide preliminary experimental support for the predicted targets and signaling pathways; however, their functional roles have yet to be established through more direct mechanistic validation.

Studies of borneol-containing formulations have now encompassed multiple CNS diseases, including AD, epilepsy, and IS, but the depth of investigation and types of evidence vary substantially among formulations. Network pharmacology and molecular docking are primarily useful for generating candidate targets and signaling pathways, whereas functional interventions, including pharmacological inhibition and gain- and loss-of-function approaches, can provide stronger support for specific mechanistic relationships. In this regard, several studies of Angong Niuhuang Pills, Suhexiang Pills, and Xingnaojing Injection have provided comparatively stronger mechanistic evidence. Nevertheless, most available studies have evaluated the formulations as a whole, their volatile fractions, or selected combinations of bioactive components, and some have employed pretreatment paradigms. Accordingly, these findings cannot be directly attributed to borneol itself and should not be considered fully equivalent to therapeutic effects under clinical treatment conditions. Overall, the available evidence supports the multi-target and multi-process pharmacological actions of borneol-containing formulations in CNS diseases. However, direct evidence remains insufficient to define the independent contribution of borneol within these formulations, its interactions with other constituent medicinal components, or the extent to which borneol contributes to the overall therapeutic effects of the complete formulation. The available evidence on borneol-containing formulations in CNS diseases is summarized in Table 7.

**TABLE 7:** Summary of borneol-containing formulations in CNS diseases.

Formulation (composition)	Source	Refs.	Disease	Model	Dose/route/timing	Main finding/mechanism	Study type	Key validation	Notes
Compound Danshen Tablets; *Salvia miltiorrhiza* + *Panax notoginseng* + borneol	Guangzhou Baiyunshan Chinese Medicine Factory	Qin et al., 2004	AD	D-galactose + IBO-induced AD rats	0.315/0.630 g/kg (crude drug), i.g., ×2 months (*in vivo*)	↓ cortical Glu; ↑ Gly/GABA	*In vivo*	HPLC amino-acid neurotransmitter assay	Early animal evidence
	Guangzhou Baiyunshan Chinese Medicine Factory	Qin et al., 2005	AD	D-galactose + IBO-induced AD rats	0.315/0.630 g/kg (crude drug), i.g., ×2 months (*in vivo*)	Improved learning/memory; ↓ brain β-AP	*In vivo*	Passive-avoidance test, β-AP radioimmunoassay	Early animal evidence
Compound Danshen Dripping Pills; *Salvia miltiorrhiza* + *Panax notoginseng* + borneol	Tasly Pharmaceutical Group	Zhao and Liu, 2020	Epilepsy	LiCl/PTZ-induced epilepsy rats	80 mg/kg, i.g., once daily ×28 days (*in vivo*)	↓ p-JAK/JAK and p-STAT3/STAT3; ↓ TNF-α/IL-6; ↓ seizure frequency and hippocampal injury	*In vivo*	Tofacitinib comparison, WB, ELISA, HE, seizure assessment	No JAK/STAT agonist or rescue validation
	NR	Huang et al., 2022	Epilepsy	Network pharmacology + molecular docking	N/A	Predicted quercetin/luteolin/tanshinone IIA–AKT1/TP53/IL-6/TNF interactions and NF-κB-related pathways	In silico	Network pharmacology, molecular docking	Computational prediction only
Angong Niuhuang Pills; *Calculus bovis* + *Bubali cornu* + *Moschus* + *Margarita* + *Cinnabaris* + *Realgar* + *Coptidis rhizoma* + *Scutellariae radix* + *Gardeniae fructus* + *Curcumae radix* + borneol	NR	Wang et al., 2024b	IS	MCAO/R rats + network pharmacology	Dose NR, p.o., ×5 days (*in vivo*)	↓ brain injury; ↑ MAPK3/SIRT1; ↓ CDC42/PARP1	In silico + *in vivo*	Network pharmacology, WB, qPCR, neurological assessment	No target-specific perturbation
	NR	Tsoi et al., 2019	IS	MCAO/R rats	257 mg/kg, p.o., single dose for efficacy; 257/514/1028 mg/kg, p.o., ×7 days for safety (*in vivo*)	↓ p47phox/iNOS/3-NT and MMP-2/MMP-9; preserved ZO-1/claudin-5; improved Bcl-2/Bax	*In vivo*	BBB/TJ assessment, oxidative/nitrosative-stress markers, MMP analysis, safety assessment	Repeated/high-dose use increased metal accumulation
	Zhangzhou Pien Tze Huang Pharmaceutical	Wang et al., 2025a	IS	MCAO/R rats	270/810 mg/kg, i.g., once daily ×3 days before MCAO; MCAO 1 h after final dose (*in vivo*)	↑ SIRT1/p62; ↓ H4K16ac, LC3B-II/I, ATG5/ATG7; ↓ apoptosis	*In vivo*	EX527 reversal, proteomics/acetylomics, IF, WB, TUNEL	Pretreatment design; SIRT1 dependence functionally supported
ANP-derived BECCs V	NR	Zhang et al., 2025b	IS	Zebrafish + MCAO mice + network pharmacology	NR	Comparable protection to ANP; PI3K/Akt/mTOR-, HIF-1α-, and FoxO3a-related regulation	In silico + *in vivo*	Network pharmacology + zebrafish + mouse MCAO; WB + behavior	BECCs system; formulation-equivalence evidence
Suhexiang Wan essential oil; *Liquidambar orientalis* + *Saussurea lappa* + *Aquilaria agallocha* + *Santalum album* + *Boswellia carteri* + *Eugenia caryophyllata* + *Cyperus rotundus* + *Styrax benzoin* + *Dryobalanops aromatica* + endo-borneol 33.3% and borneol 21.3%	NR	Jeon et al., 2011	AD	Aβ_1-42_-injected mice + Aβ_1-42_-stimulated SH-SY5Y cells	2 g essential oil/cage; inhalation 3 h/session, twice daily ×14 days before Aβ_1-42_ injection (*in vivo*); 1/10/100 μg/mL pretreatment before Aβ_1-42_ exposure (*in vitro*)	↑ Nrf2/HO-1; ↓ ROS/apoptosis and p38/JNK/Tau phosphorylation; improved memory	*In vivo* + *in vitro*	ROS assay, Hoechst/MTT, WB, Y-maze/passive avoidance	Modified volatile fraction; preventive design
Suhexiang Pills; *Styrax* + *Benzoinum* + *borneol* + *Bubali cornu* + *Moschus* + *Santali albi lignum* + *Aquilariae lignum resinatum* + *Caryophylli flos* + *Cyperi rhizoma* + *Aucklandiae radix* + *Olibanum* + *Piperis longi fructus* + *Atractylodis macrocephalae rhizoma* + *Chebulae fructus* + *Cinnabaris*	NR	Zhang et al., 2024	IS	Acute cerebral infarction rats	2.5/5/10 mL/kg, i.g., once daily ×2 weeks (*in vivo*)	↑ cAMP/p-PKA; ↓ TNF-α/IL-1β, Bax, and cleaved caspase-3	*In vivo*	SQ22536 reversal, WB, ELISA, TTC, neurological assessment	cAMP/PKA involvement functionally supported
	Beijing Tongrentang	Li et al., 2025b	IS	OGD/R primary cortical neurons	0.91 g/kg, p.o., once daily ×7 days to donor mice; 10% drug-containing serum during reoxygenation (*in vitro*)	↑ miR-24-3p/Nrf2/HO-1; ↓ Keap1, inflammation, and apoptosis	*In vitro*	miR-24-3p inhibitor reversal, qPCR, WB, CCK-8, dual-luciferase assay	Medicated-serum design; no ShxP-treated stroke animals
Xingnaojing Injection; *Moschus* + *Curcuma aromatica rhizome* + borneol + *Gardeniae fructus*	NR	Wang et al., 2020a	AD	Microarray analysis + network pharmacology	N/A	Predicted HTR2A/ADRA2A and neuroactive ligand–receptor interaction	In silico	Microarray integration, network pharmacology, qRT-PCR of candidate genes	No direct XNJ target validation
	NR	Liu et al., 2020	AD	Aβ_1-42_-induced AD mice	Dose NR, i.p., timing NR (*in vivo*)	↑ AKT/mTOR-related signaling, NMDAR-mediated transmission, dendritic spine density, and LTP	*In vivo*	Rapamycin reversal, electrophysiology/LTP, WB, behavioral tests	AKT/mTOR involvement functionally supported
	NR	Qu et al., 2019	IS	MCAO/R rats	Dose NR, i.p., timing NR (*in vivo*)	↓ NLRP3; preserved ZO-1/occludin; ↓ BBB leakage	*In vivo*	EB permeability, WB, ELISA, neurological assessment	No NLRP3-specific perturbation
	NR	Zhang et al., 2020	IS	MCAO/R rats	Dose NR, i.p., timing NR (*in vivo*)	↑ SIRT1; ↓ inflammatory responses	*In vivo*	EX527 reversal, WB, qPCR, ELISA, neurological assessment	SIRT1 involvement functionally supported
	NR	Liu et al., 2022	IS	Network pharmacology + tMCAO mice	NR	↓ TLR4/MyD88/NF-κB; improved BBB permeability; altered gut-barrier/microbiota parameters	In silico + *in vivo*	Network pharmacology, 16S rRNA sequencing, WB, BBB assessment	No target-specific perturbation
	Henan Tiandi Pharmaceutical	Cao et al., 2024	IS	MCAO rats	10/15 mL/kg, i.p., once daily ×5 days before MCAO/R + one dose at reperfusion (*in vivo*)	↓ HMGB1/NLRP3/NF-κB, caspase-1, and GSDMD	*In vivo*	ELISA, beam-walking test, neurological scoring	Pretreatment design; marker-based pyroptosis evidence
	NR	Dong et al., 2024	IS	MCAO/R rats + OGD/R neurons	2 mL/kg, i.p., 2 h post-modeling (*in vivo*); dose/timing NR (*in vitro*)	↓ neuronal Ca^2+^ overload and ER stress; ↓ apoptosis/inflammation	*In vivo* + *in vitro*	Ca^2+^ assessment, WB, qPCR, TUNEL, neurological assessment	No ER-stress-specific perturbation
Compound Shexiang Injection; *Moschus* + borneol + *Curcumae radix* + *Pogostemonis herba* + *Acori tatarinowii rhizoma* + menthol	NR	Li et al., 2023	IS	OGD/R HT-22 cells + network pharmacology	0.0001/0.001/0.01%; 8-h pretreatment + 12-h OGD + 12-h reoxygenation (*in vitro*)	Predicted PI3K/Akt, Rap1, and MAPK pathways; trends toward ↑ PIK3CA/MAPK3 and ↓ SRC mRNA	In silico + *in vitro*	Network pharmacology, molecular docking, RT-qPCR, CCK-8	Non-significant expression/viability trends; functional roles untested

Study type indicates the original experimental design; Key validation summarizes the principal experimental approaches; Notes highlight major considerations relevant to mechanistic interpretation. All studies evaluated complete formulations, formulation-derived volatile fractions, selected component combinations, or computationally reconstructed systems; none isolated the independent contribution of borneol using a borneol-only or borneol-depleted control. Accordingly, the observed effects should be attributed to the corresponding formulation/intervention rather than to borneol itself. ↑/↓ indicate increase/decrease; N/A, not applicable; NR, not reported.

## 5 Pharmacokinetic characteristics and brain exposure of borneol

The pharmacokinetic characteristics of borneol provide an important basis for evaluating its translational potential in CNS applications. Zhao et al. (2012) compared the pharmacokinetic profiles of borneol following intravenous, intranasal, and oral administration in mice. The absolute bioavailability after intranasal administration was 90.7%, approximately twice that achieved after oral administration (43.0%). The relative brain-targeting coefficients after intranasal and oral administration were 68.37% and 38.40%, respectively. Moreover, the times to peak concentration in plasma and brain tissue after intranasal administration were 3.0 and 3.4 min, respectively, both shorter than those observed after oral administration. These findings suggest that intranasal administration may facilitate rapid systemic absorption and brain exposure of borneol. More recently, borneol has also been incorporated into drug-delivery systems as a permeability-enhancing component (Cheng et al., 2026). In intranasal delivery, borneol-mediated modulation of nasal mucosal barrier permeability has been reported to facilitate nose-to-brain transport of therapeutic agents (Wu F. et al., 2023a; Wang G. et al., 2024a). In nanocarrier-based delivery systems, borneol-modified polymeric micelles and brain-targeted nucleic acid delivery platforms have likewise shown potential to improve delivery efficiency to the brain (Wang Y. et al., 2025c; Cheng et al., 2026).

Beyond the influence of administration routes, brain exposure to borneol also exhibits distinct temporal and regional distribution characteristics. Li et al. (2012) showed that borneol was detectable in brain tissue as early as 5 min after oral administration of natural borneol in mice, reaching a maximum brain concentration of 86.52 μg/g at approximately 1 h, indicating rapid penetration of borneol into the brain. However, this study used a relatively high animal dose of 1.2 g/kg natural borneol, and therefore the observed brain concentration cannot be directly considered equivalent to therapeutic exposure in humans. Borneol distribution within the brain also appears to be region-dependent. Using brain microdialysis, Yu et al. (2013) found that borneol concentrations were highest in the cerebral cortex, followed by the hippocampus and hypothalamus, whereas lower levels were detected in the striatum. However, whether this regional distribution is directly associated with selective pharmacological targeting remains insufficiently established.

The *in vivo* metabolism of borneol may also influence its pharmacological effects and safety profile. Cheng et al. (2013) investigated the pharmacokinetic characteristics of natural borneol and synthetic borneol after intravenous administration in rats and found that plasma concentrations exhibited biphasic elimination, with an elimination half-life of approximately 8 h. Camphor was detected as a metabolite, and isoborneol showed a greater tendency to undergo conversion to camphor than borneol. These findings suggest that borneol from different sources or with different stereochemical configurations may exhibit distinct metabolic characteristics *in vivo*.

Human pharmacokinetic data for borneol remain limited. A phase I clinical study of d-borneol showed that single and repeated intravenous infusions of 10–40 mg SPT-07A resulted in a terminal elimination half-life ranging from approximately 3.85–8.93 h. Following administration every 12 h for approximately 4 days, steady-state conditions were achieved with limited accumulation. The compound was primarily eliminated in the urine as glucuronide conjugates, with a total urinary recovery rate of approximately 84.69% (Wang W. et al., 2020). However, this study only reported plasma pharmacokinetic parameters and did not measure brain concentrations of borneol. Therefore, the effective brain exposure required for therapeutic effects in CNS diseases remains undetermined.

Although current studies suggest that borneol can rapidly reach brain tissue, key pharmacokinetic parameters, including effective brain concentrations, human bioavailability, and elimination half-life, remain insufficiently characterized. Differences among borneol stereoisomers in absorption, distribution, metabolism, and elimination, as well as potential tissue accumulation following long-term administration and the biological activities of major metabolites and their relationships with pharmacological effects, remain to be elucidated. Furthermore, reliable correlations between doses used in animal studies and clinically relevant exposure levels have not yet been established, and current clinical studies lack data regarding borneol exposure in human brain tissue or cerebrospinal fluid. Therefore, future studies should integrate brain exposure profiles, metabolite profiling, pharmacodynamic biomarkers, and toxicity endpoints to establish robust exposure–response relationships.

## 6 Clinical evidence and therapeutic applications

Clinical evidence related to borneol in CNS diseases has primarily focused on IS, with edaravone dexborneol combination therapy, consisting of edaravone and d-borneol, representing the most extensively investigated formulation. Several multicenter, randomized, double-blind controlled trials involving more than one thousand patients with acute ischemic stroke demonstrated that edaravone dexborneol combination therapy achieved superior 90-day functional outcomes compared with edaravone monotherapy (Xu et al., 2019). A systematic review and meta-analysis updated in 2026, including 13 studies and 7,846 patients, further supported an association between edaravone dexborneol therapy and favorable 90-day functional outcomes. However, dose-related safety signals, including hyperhomocysteinemia and hypokalemia, were also reported (Sabet et al., 2026).

Importantly, these findings support the clinical benefits of the combination formulation rather than providing direct evidence for the independent therapeutic efficacy of d-borneol. Clinical evidence for borneol in AD, PD, epilepsy, and glioma remains insufficient, with current knowledge derived predominantly from cellular and animal studies. Furthermore, some cerebral ischemia and formulation-based studies have employed pretreatment paradigms before disease induction, which do not fully correspond to the therapeutic timing of acute ischemic stroke in clinical practice. Therefore, protective effects observed under preventive administration conditions cannot be directly extrapolated to therapeutic efficacy. Future studies should distinguish among different treatment windows, including preventive intervention, acute treatment, and recovery-phase administration.

## 7 Safety and toxicological evidence

Borneol preparations of different origins and stereochemical configurations may exhibit distinct safety profiles, and available evidence suggests that natural borneol, l-borneol, d-borneol, and synthetic borneol differ to some extent in both pharmacological activity and toxicity (Ma et al., 2023a). Direct human safety data for d-borneol are currently derived mainly from the phase I study of SPT-07A. In this study, 36 healthy volunteers received single and repeated intravenous doses of 10–40 mg, and all reported adverse events were mild to moderate and resolved spontaneously, providing preliminary evidence of short-term tolerability within this dose range. However, the small sample size, enrollment of healthy participants, and limited observation period preclude definition of a long-term therapeutic safety window (Wang W. et al., 2020b).

With respect to target-organ toxicity, Liu et al. (2023) found that exposure of zebrafish larvae to 300–500 μM borneol induced reductions in liver size, impaired hepatic function, and intrahepatic lipid accumulation, accompanied by changes related to oxidative stress, endoplasmic reticulum stress, and autophagy. Li B. et al. (2025a) further reported that high-dose borneol exposure (500 μM) caused morphological abnormalities, cardiotoxicity, neurobehavioral alterations, and abnormal development of dopaminergic neurons in zebrafish, whereas no overt nephrotoxicity was observed. By contrast, a 30-day repeated-dose study published in 2026 reported no obvious toxic effects in rats administered borneol at doses of up to 270 mg/kg/day, suggesting a potential safety margin under these specific experimental conditions. Nevertheless, these findings cannot substitute for long-term chronic toxicity assessment (Luo et al., 2026). Evidence from multicomponent preparations should be interpreted separately from that for purified borneol. An acute toxicity study of essential oil from the borneol chemotype of *C. camphora*, containing approximately 18% borneol, reported oral median lethal dose (LD50) values of 2,749 mg/kg in female mice and 5,081 mg/kg in male mice, corresponding to low-toxicity and practically non-toxic categories, respectively. Preparations at concentrations below 50% produced no detectable skin or ocular irritation (Xiao et al., 2021). However, because this essential oil is a multicomponent preparation, these toxicological findings cannot be directly attributed to borneol alone. A clinical risk assessment of 121 Chinese patent medicines containing borneol classified 13, 50, and 58 products as high-, moderate-, and low-risk, respectively. High-risk products were primarily associated with the concomitant presence of potentially toxic medicinal ingredients or a greater number of reported adverse reactions, highlighting the importance of rational prescribing, appropriate treatment duration, and risk management in the clinical use of borneol-containing formulations (Wei et al., 2026).

Overall, the safety profile of borneol may be influenced by dose, route of administration, source, and stereochemical configuration. However, the available evidence remains insufficient to define a clear safety dose range for the treatment of CNS diseases, and data on long-term use, repeated administration, and special populations are still limited. The potential impact of borneol-mediated modulation of barrier permeability on the brain exposure of co-administered drugs also warrants further investigation. Future studies should therefore undertake rigorous dose–exposure–toxicity assessments to better characterize the therapeutic safety window of borneol.

## 8 Knowledge gaps and future directions

Although accumulating evidence has revealed a potential pharmacological basis for borneol in CNS diseases through the modulation of brain barriers, neuroinflammation and oxidative stress, neurotransmitter homeostasis, cellular injury and death, and the clearance of pathological substances, the overall evidence base remains dominated by cellular and animal studies. Substantial heterogeneity also exists across studies with respect to borneol source, stereochemical configuration, purity, dose, route of administration, disease model, and timing of intervention. Consequently, current findings support the therapeutic potential of borneol in CNS disorders but remain insufficient to establish a consistent, reproducible, and clinically translatable mode of action. Several major knowledge gaps therefore need to be addressed.

### 8.1 Defining pharmacological differences and stereoselectivity among borneol preparations

Whether different forms of borneol are pharmacologically equivalent has not been clearly established. Natural borneol, synthetic borneol, l-borneol, d-borneol, and Aipian may differ in chemical composition, stereochemical configuration, *in vivo* disposition, and safety profiles. However, many primary studies provide insufficient information regarding the source, purity, and stereochemical identity of the borneol preparation used, thereby limiting direct comparisons and evidence synthesis across studies.

Future investigations should therefore incorporate standardized physicochemical characterization of borneol preparations and perform head-to-head comparisons of different sources and stereochemical forms under matched disease models, doses, and administration conditions. Such studies should determine whether pharmacological efficacy, brain exposure, metabolism, and toxicity exhibit stereoselectivity. It will also be important to define disease indications and molecular targets for which specific forms of borneol may offer relative advantages, thereby providing a basis for more precise and differentiated therapeutic use.

### 8.2 Strengthening causal mechanistic validation and multilevel integration

The strength of mechanistic evidence varies considerably across the current literature. Some studies have used pathway inhibitors, gain- and loss-of-function approaches, or other functional interventions to support the involvement of specific signaling axes. However, many proposed mechanisms are still inferred primarily from parallel changes in protein expression, inflammatory mediators, behavioral outcomes, or histopathological indices, whereas network pharmacology and molecular docking largely provide candidate targets and pathway predictions.

Future studies should therefore place greater emphasis on causal validation using genetic knockdown or knockout, selective pharmacological inhibitors or agonists, and rescue experiments. Such approaches are essential to distinguish genuine pathway dependence from molecular changes that merely accompany the observed phenotype. Moreover, current research has largely focused on individual signaling pathways or restricted sets of molecular markers, providing only a fragmented view of the broader regulatory actions of borneol. Integration of transcriptomics, proteomics, metabolomics, single-cell sequencing, and spatial omics may help define cell type-, brain region-, and disease stage-specific regulatory networks. These systems-level observations should subsequently be linked to functional validation of key nodes and interactions, allowing the field to move beyond associative molecular profiling toward causal and network-level mechanistic understanding.

### 8.3 Resolving disease- and context-dependent actions

The pharmacological actions of borneol are strongly dependent on disease and pathological context. In ischemia-associated pathological BBB disruption, borneol predominantly appears to preserve or restore barrier integrity. By contrast, in drug-delivery and glioma settings, modulation of BBB or BTB permeability and transport processes can increase drug exposure in the brain or tumor tissue. A similar context dependence is evident for oxidative stress: attenuation of oxidative injury is generally associated with neuroprotection in cerebral ischemia and neurodegenerative disorders, whereas enhancement of ROS-mediated damage may increase the chemosensitivity of glioma cells. Autophagy may likewise exert distinct effects depending on disease stage, cellular context, and therapeutic setting.

Future studies should therefore avoid treating increased BBB permeability, antioxidant activity, or enhanced autophagy as uniformly beneficial outcomes. Instead, the direction and magnitude of these effects should be evaluated in relation to dose, timing of intervention, disease stage, and pathological state. Particular attention should be given to the magnitude, reversibility, and duration of borneol-induced changes in barrier permeability, as well as the possibility that enhanced barrier permeability could inadvertently increase brain exposure to non-target drugs or potentially harmful circulating substances. Defining these boundaries will be essential for establishing disease- and treatment-specific therapeutic windows.

### 8.4 Establishing dose–exposure–response relationships and improving clinical translation

Common experimental paradigms in the current literature, including pretreatment, high-dose administration, and non-standard routes of delivery, do not always reflect clinically relevant treatment conditions. Although borneol has been shown to reach brain tissue rapidly and to exhibit measurable brain exposure and stereochemistry-dependent pharmacokinetic differences, human bioavailability, therapeutically effective brain concentrations, clinically equivalent doses, and the biological activities of major metabolites remain insufficiently characterized. Reliable quantitative relationships between doses used in animal studies and clinically relevant human exposure have also not been established.

Future studies should therefore define quantitative relationships across the continuum of dose, plasma exposure, brain exposure, pharmacological response, and toxicity. This will require integrated pharmacokinetic/pharmacodynamic studies incorporating brain concentrations, metabolite profiles, pharmacodynamic biomarkers, and toxicity endpoints. Preventive administration, acute treatment after disease onset, and interventions during the recovery phase should also be investigated separately, rather than extrapolating protective effects obtained from pretreatment paradigms directly to therapeutic efficacy.

High-quality clinical evidence remains limited, particularly for borneol administered as an independent therapeutic agent. Beyond combination products such as edaravone dexborneol, rigorously designed clinical studies of borneol itself are scarce. Large, multicenter randomized controlled trials with standardized efficacy and safety endpoints are therefore needed. Long-term and repeated administration, as well as use in special populations, also require rigorous evaluation. Particular attention should be paid to potential neurotoxicity, hepatic and other target-organ toxicities, tissue accumulation, and the definition of a long-term therapeutic safety window.

### 8.5 Clarifying the independent contribution of borneol within multi-component formulations

Most studies of borneol-containing formulations have evaluated complete formulations, volatile fractions, or selected combinations of bioactive components. Borneol-depleted formulations, borneol add-back experiments, and parallel comparisons with purified borneol are rarely included. Consequently, the independent contribution of borneol within these formulations remains unclear, as does the extent to which borneol interacts synergistically, additively, or antagonistically with other medicinal or bioactive components. Likewise, findings obtained with formulations such as Angong Niuhuang Pills, Suhexiang Pills, and Xingnaojing Injection cannot be directly extrapolated to borneol itself.

Future studies should therefore use parallel experimental designs incorporating the complete formulation, a borneol-depleted formulation, borneol add-back conditions, and borneol administered alone, with the source, purity, stereochemical configuration, and dose of borneol clearly defined. Integration of pharmacokinetic and pharmacodynamic analyses may further enable quantitative estimation of the contribution of borneol to the overall effects of complex formulations. Ultimately, standardized preclinical studies and rigorously designed randomized controlled trials will be required to determine the genuine clinical efficacy, long-term safety, and most appropriate target populations for borneol and borneol-containing formulations.

## 9 Conclusion

Overall, borneol shows multifaceted pharmacological potential in CNS diseases through the modulation of brain barriers, neuroinflammation and oxidative stress, neurotransmitter homeostasis, cellular injury and death, and the clearance of pathological substances. However, its effects are strongly disease- and context-dependent, and the current evidence remains limited by insufficient causal validation, scarce high-quality clinical data, and a lack of robust long-term safety evaluation. Clarifying the pharmacological differences among borneol preparations, establishing dose–exposure–response relationships, and defining the independent contribution of borneol within complex formulations will be essential for determining its genuine therapeutic value and rational clinical application in CNS diseases.
